# RstA Is a Major Regulator of Clostridioides difficile Toxin Production and Motility

**DOI:** 10.1128/mBio.01991-18

**Published:** 2019-03-12

**Authors:** Adrianne N. Edwards, Brandon R. Anjuwon-Foster, Shonna M. McBride

**Affiliations:** a Department of Microbiology and Immunology, Emory Antibiotic Resistance Center, Emory University School of Medicine, Atlanta, Georgia, USA; b Department of Microbiology and Immunology, University of North Carolina at Chapel Hill School of Medicine, Chapel Hill, North Carolina, USA; University of Pittsburgh School of Medicine

**Keywords:** *Clostridium*, *Clostridium difficile*, RNPP, RRNPP, TcdA, TcdB, helix-turn-helix, motility, spore, sporulation, toxin, transcriptional regulator

## Abstract

Clostridioides difficile infection (CDI) is a toxin-mediated diarrheal disease. Several factors have been identified that influence the production of the two major C. difficile toxins, TcdA and TcdB, but prior published evidence suggested that additional unknown factors were involved in toxin regulation. Previously, we identified a C. difficile regulator, RstA, that promotes sporulation and represses motility and toxin production. We observed that the predicted DNA-binding domain of RstA was required for RstA-dependent repression of toxin genes, motility genes, and *rstA* transcription. In this study, we further investigated the regulation of toxin and motility gene expression by RstA. DNA pulldown assays confirmed that RstA directly binds the *rstA* promoter via the predicted DNA-binding domain. Through mutational analysis of the *rstA* promoter, we identified several nucleotides that are important for RstA-dependent transcriptional regulation. Further, we observed that RstA directly binds and regulates the promoters of the toxin genes *tcdA* and *tcdB*, as well as the promoters for the *sigD* and *tcdR* genes, which encode regulators of toxin gene expression. Complementation analyses with the Clostridium perfringens RstA ortholog and a multispecies chimeric RstA protein revealed that the C. difficile C-terminal domain is required for RstA DNA-binding activity, suggesting that species-specific signaling controls RstA function. Our data demonstrate that RstA is a transcriptional repressor that autoregulates its own expression and directly inhibits transcription of the two toxin genes and two positive toxin regulators, thereby acting at multiple regulatory points to control toxin production.

## Abstract (precis)

Clostridioides difficile is an anaerobic, gastrointestinal pathogen of humans and other mammals. C. difficile produces two major toxins, TcdA and TcdB, which cause the symptoms of the disease, and forms dormant endospores to survive the aerobic environment outside the host. A recently discovered regulatory factor, RstA, inhibits toxin production and positively influences spore formation. Herein, we determine that RstA directly binds its own promoter DNA to repress its own gene transcription. In addition, our data demonstrate that RstA directly represses toxin gene expression and gene expression of two toxin gene activators, TcdR and SigD, creating a complex regulatory network to tightly control toxin production. This study provides a novel regulatory link between C. difficile sporulation and toxin production. Further, our data suggest that C. difficile toxin production is regulated through a direct, species-specific sensing mechanism.

## INTRODUCTION

Clostridioides difficile infection (CDI) is a nosocomial and community-acquired gastrointestinal disease that affects individuals with dysbiotic gut microbiota, which commonly occurs after antibiotic treatment (1, 2). Clinical outcomes range from mild diarrhea to severe disease symptoms, including sepsis and death (1). The two glycosylating exotoxins, TcdA and TcdB, elicit CDI symptoms and are indispensable for C. difficile virulence (3). Environmental and intracellular signals, including nutrient availability and metabolic cues, strongly influence toxin production (4–7). There are numerous identified C. difficile factors that control toxin gene expression in response to these signals (8–12); however, the regulatory pathways and molecular mechanisms that directly control toxin gene expression are not fully understood (13).

Our previous work identified a novel regulator, RstA, which depresses C. difficile toxin production and motility (14). RstA inhibits transcription of the toxin genes *tcdA* and *tcdB*, the toxin-specific sigma factor, *tcdR*, and the flagellum-specific sigma factor, *sigD*, which is essential for motility and directs *tcdR* expression (11, 12, 14–16). In addition to repressing motility and toxin production, RstA positively influences C. difficile spore formation, which is critical for the survival of the bacterium outside of the host and for transmission from host to host, indicating that RstA regulates diverse phenotypes important for C. difficile pathogenesis. An *rstA* mutant exhibits increased toxin gene expression *in vivo* and is more virulent in the hamster model of CDI, demonstrating the impact RstA has on pathogenesis (14).

The predicted secondary structure of RstA reveals three apparent domains: an N-terminal conserved helix-turn-helix DNA-binding domain, followed by a series of multiple tetratricopeptide repeat (TPR) domains comprising a putative Spo0F-like protein-binding domain, and a C-terminal putative quorum-sensing-like domain (14). These characteristic features place RstA in the RRNPP (Rap/Rgg/NprR/PlcR/PrgX; formerly RNPP) family of proteins. RRNPP proteins are prevalent in Gram-positive organisms and regulate competence, sporulation, toxin production, and other important survival and virulence phenotypes (17–19). The DNA-binding or protein-binding activity of RRNPP proteins are controlled by the direct binding of small, quorum-sensing peptides (19). The precursor proteins encoding the quorum-sensing peptides are often adjacent to the regulatory RRNPP protein and are translated, exported, processed, and reinternalized at high cell densities (20–25). In addition, RRNPP proteins often autoregulate their own expression, as is observed for RstA (14). The presence of these conserved domains within RstA provides insight into how RstA may regulate C. difficile toxin production, motility, and sporulation.

To better understand the regulatory impact RstA exerts on C. difficile toxin production and sporulation, we examined the function of the conserved DNA-binding domain. Our previous study (14) had shown that the DNA-binding domain is required for RstA-dependent regulation of *rstA* expression and toxin gene expression but is expendable for sporulation regulation. Here, we demonstrate that RstA directly binds to its promoter via an imperfect inverted repeat and that it directly binds the *sigD* and toxin gene promoters. Further, our data demonstrate that RstA and SigD independently control toxin expression, creating a multitiered regulatory pathway by which RstA represses toxin production. Finally, we show that the Clostridium perfringens *rstA* ortholog does not complement toxin production or sporulation in a C. difficile *rstA* mutant. However, a chimeric RstA protein containing the C. perfringens DNA-binding domain and the C. difficile Spo0F-binding and quorum-sensing-binding domains restores sporulation and represses toxin production, providing evidence that the ability to respond to species-specific signaling is necessary for RstA DNA-binding activity.

## RESULTS

### RstA autoregulates its gene transcription via an inverted repeat overlapping the promoter.

Our previous work provided preliminary genetic evidence that the N-terminal putative helix-turn-helix DNA-binding domain was necessary for inhibition of toxin gene expression but was dispensable for sporulation initiation (14). However, further work with the recombinant His-tagged RstA proteins revealed that the constructs were expressed at low levels and were not detected by Western blotting of C. difficile lysates (data not shown). We created a new series of tagged proteins, possessing the 3×FLAG tag on the C-terminal end and found that these were stably expressed and easily detected in C. difficile *rstA*::*erm* lysates (see Fig. S2A in the supplemental material). Corroborating our previous data (14), expression of the wild-type RstA, the full-length FLAG-tagged RstA, and the truncated RstAΔHTH-FLAG-tagged allele complemented sporulation in the *rstA* mutant (Fig. S2B). As previously observed (14), only full-length RstA restored toxin production to wild-type levels in the *rstA* background (Fig. S2C and D), confirming that the helix-turn-helix motif within the DNA-binding domain is essential for RstA-dependent control of toxin production.

**FIG 1:**
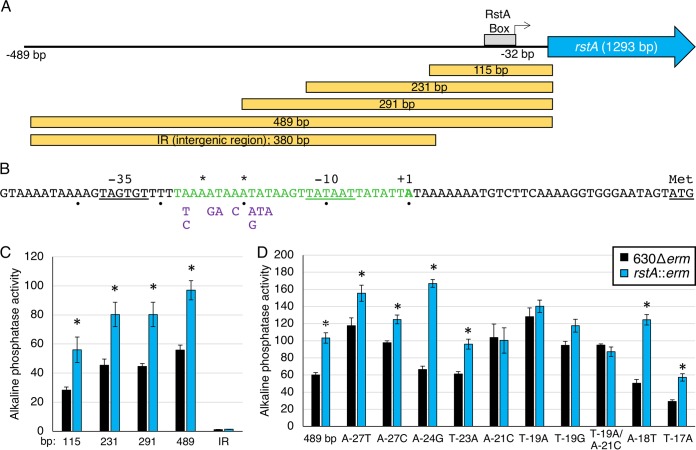
RstA controls its gene expression through an inverted repeat sequence overlapping the *rstA* promoter. (A) A schematic of the *rstA* promoter region denoting the general location of the putative RstA box, the transcriptional start (32 bp upstream from the start codon; represented by the bent arrow), and the *rstA* open reading frame (not to scale). The yellow boxes indicate the locations and sizes of promoter fragments constructed for the *phoZ* reporter fusions in panel C. (B) The *rstA* promoter, marked by +1, overlaps a 29-bp imperfect inverted repeat (shown in green). The asterisks above the sequence mark the mismatched nucleotides within the inverted repeat. The −10 and −35 consensus sequences and the ATG start codon are underlined. The nucleotides below the sequence represent the substitutions tested in panel D. (C and D) Alkaline phosphatase (AP) activity of the P*rstA*::*phoZ* reporter fusions of various lengths, including the upstream intergenic region (IR) (−489 bp to −112 relative to the translational start) of *rstA* (C) (P*rstA*_115_ [MC979/MC980]), P*rstA*_231_ [MC1010/MC1011], P*rstA*_291_ [MC1012/MC1013], P*rstA*_489_ [MC773/MC774], P*rstA*_IR_ [MC1008/MC1009]) or of the full-length P*rstA*::*phoZ* promoter with various nucleotide substitutions (D) (P*rstA*_489_ [MC773/MC774], P*rstA*_A-27T_ [MC830/MC831], P*rstA*_A-27C_ [MC856/MC857], P*rstA*_A-24G_ [MC858/MC859], P*rstA*_T-23A_ [MC832/MC833], P*rstA*_A-21C_ [MC860/MC861], P*rstA*_T-19A_ [MC834/MC835], P*rstA*_T-19G_ [MC862/MC863], P*rstA*_T-19A/A-21C_ [MC1433/1434], P*rstA*_A-18T_ [MC836/MC837], P*rstA*_T-17A_ [MC838/MC839]) in strain 630Δ*erm* and the *rstA*::*erm* mutant (MC391), respectively, grown on 70:30 sporulation agar at H_8_. The means ± standard errors of the means for four biological replicates are shown. Values that are significantly different (*P < *0.05) by Student’s *t* test from the activity observed for the 630Δ*erm* parent strain for each promoter construct are indicated by an asterisk.

We hypothesized that RstA directly binds to DNA to control toxin gene expression and transcription of additional target genes. This interaction is predicted to occur via the putative DNA-binding domain, as observed for other RRNPP transcriptional regulators (26–28). Additionally, we previously observed that *rstA* expression remains relatively unchanged throughout growth in multiple conditions and that *rstA* transcription is increased in an *rstA* mutant (14), suggesting that expression of *rstA* may be autoregulated. To determine whether RstA is DNA-binding protein, we first defined the *rstA* promoter region and probed the DNA-binding capability of RstA within its own promoter. The transcriptional start of *rstA* was identified at −32 bp upstream from the translational start using 5′ RACE. Corresponding σ^A^ −10 and −35 consensus sequences were detected immediately upstream of this transcriptional start site (Fig. 1A and B). To verify the mapped promoter and to determine whether any additional promoters are present that drive *rstA* transcription, a series of promoter fragments fused to the *phoZ* reporter gene was created, and alkaline phosphatase (AP) activity was measured in the 630Δ*erm* and *rstA*::*erm* mutants. As previously observed, the full-length 489-bp *rstA* promoter fragment exhibited a 1.8-fold increase in activity in the *rstA* mutant compared to the parent strain, indicating RstA-dependent repression (Fig. 1C) (14). The truncated promoter fragments, P*rstA*_291_ and P*rstA*_231_, produced similar fold changes in activity in the *rstA* mutant and parent strains, as observed for the full-length promoter. However, reporter activity was lower in the P*rstA*_115_ fragment compared to the longer fragments, suggesting that an enhancer sequence or an additional RstA-independent transcriptional activator is located between −231 bp to −115 bp upstream of the *rstA* open reading frame. A promoter fragment reporter fusion containing 380 bp of sequence upstream from the mapped *rstA* promoter [from −489 bp to −112 bp; intergenic region (IR) in Fig. 1A] was inactive, indicating that an additional promoter is not located within this region. We also tested whether RstA-dependent repression of the full-length P*rstA* reporter could be complemented. We expressed the *rstA-*FLAG construct from the nisin-inducible promoter, *cprA* (29), divergently from the P*rstA*::*phoZ* construct on the same plasmid in the *rstA*::*erm* background. P*rstA* reporter activity was reduced in a dose-dependent manner relative to the amount of nisin added to the medium (Fig. S3), further confirming the autoregulatory effect RstA exerts on its own expression. Altogether, the data demonstrate that the mapped σ^A^-dependent promoter drives *rstA* expression and that RstA can repress transcription from this promoter.

The results obtained from the promoter-reporter fusions suggested that RstA binding was likely to occur within the 115 bp upstream of the translational start site. A 29-bp imperfect inverted repeat was identified within the predicted P*rstA* −10 consensus sequence, suggesting a possible regulatory binding site within this region (Fig. 1B). To determine whether this sequence serves as an RstA recognition site, we created a series of single nucleotide substitutions within the inverted repeat in the 489-bp P*rstA* reporter fusion, avoiding conserved residues required for RNAP-holoenzyme recognition (30). Most of the single nucleotide substitutions did not significantly alter reporter activity compared to the wild-type P*rstA* reporter (Fig. 1D). However, nucleotide substitutions in two positions, A-21 and T-19, abolished RstA repression in the parent strain, increasing reporter activity to match that of the *rstA*::*erm* mutant. These data suggest that the A-21 and T-19 nucleotides are important for RstA binding to the *rstA* promoter.

### RstA inhibits toxin and motility gene transcription.

Regulatory control of toxin gene expression in C. difficile involves multiple sigma factors and transcriptional regulators, which ensure that toxin production occurs in the appropriate environmental conditions (13). Our previous work (14) demonstrated that an *rstA*::*erm* mutant has increased transcription of the C. difficile toxin genes, *tcdA* and *tcdB*, the toxin-specific sigma factor, *tcdR*, and the flagellum-specific sigma factor, *sigD*, which is required for motility and directs *tcdR* transcription (11, 12). To determine whether RstA is involved directly in repressing transcription of these genes, we first constructed *phoZ* reporter fusions with the promoter regions for each gene and examined RstA-dependent transcriptional activity.

**FIG 2:**
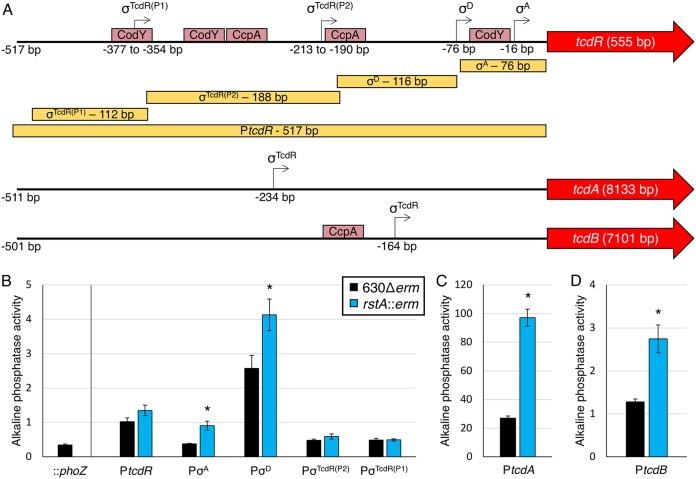
RstA inhibits toxin gene expression. (A) A schematic of the promoter regions of *tcdR*, *tcdA*, and *tcdB* denoting the relative locations of the transcriptional start sites experimentally demonstrated (12, 32–34) and the open reading frames of all three genes (not drawn to scale). Pale red boxes approximate CodY- and CcpA-binding sites within the toxin gene promoters (8, 9, 36). The yellow boxes indicate the locations and sizes of the promoter fragments constructed for the *phoZ* reporter fusions in panels B to D. Alkaline phosphatase (AP) activity of the P*tcdR*::*phoZ* reporter fusions of various lengths (B) (promoterless *phoZ* [MC448], P*tcdR*_σA_ [MC1285/MC1286], P*tcdR*_σD_ [MC1145/MC1146], P*tcdR*_σTcdR(P2)_ [MC1147/MC1148], and P*tcdR*_σTcdR(P1)_ [MC1149/MC1150]) and the P*tcdA*::*phoZ* (C) (−511 bp to −1 bp upstream of transcriptional start; MC1249/MC1250) or P*tcdB*::*phoZ* (D) (−531 bp to −31 bp upstream of transcriptional start [MC1251/MC1252]) reporter fusions in strain 630Δ*erm* and the *rstA*::*erm* mutant (MC391) grown in TY medium (pH 7.4) at H_24_. The means and standard errors of the means for four biological replicates are shown. *, *P < *0.05, using Student’s *t* test compared to the activity observed in the 630Δ*erm* parent strain for each promoter construct.

The *tcdR* promoter region contains four identified independent promoter elements: a σ^A^-dependent promoter (−16 bp from the translational start), a σ^D^-dependent promoter (−76 bp from the translational start), and two putative σ^TcdR^ promoters farther upstream (Fig. 2A) (11, 12, 31–33). Expression of the *tcdR* gene is relatively low in C. difficile (11, 32, 34), at least in part due to repression by CodY and CcpA binding throughout the *tcdR* promoter region under nutrient-rich conditions (8, 9, 33, 35, 36). We examined each of the promoter elements within P*tcdR* to determine whether RstA affects transcription from these promoters. A series of reporter fusions was created for each of the promoter elements, which were examined in the *rstA*::*erm* mutant and parent strain, and activity was measured after 24 h of growth in TY medium (Fig. 2A). A full-length 517-bp P*tcdR*::*phoZ* reporter and the two σ^TcdR^-dependent promoter fusions exhibited similar low reporter activities in the parent and *rstA* strains (Fig. 2B). However, increased reporter activity was observed in the *rstA* mutant for the individual σ^A^-dependent and σ^D^-dependent promoter fusions. These results indicate that RstA impacts the function of these promoter elements and contributes to repression of *tcdR* transcription.

We also examined RstA-dependent regulation of *tcdA* and *tcdB* transcription, both of which are expressed solely from σ^TcdR^-dependent promoters (Fig. 2A) (34, 37, 38). P*tcdA* reporter activity was increased 3.6-fold and P*tcdB* activity was 2.1-fold greater in the *rstA* strain compared to the parent (Fig. 2C and D). Altogether, these data indicate that RstA represses toxin gene transcription at the individual gene level and through repression of *tcdR*.

**FIG 3:**
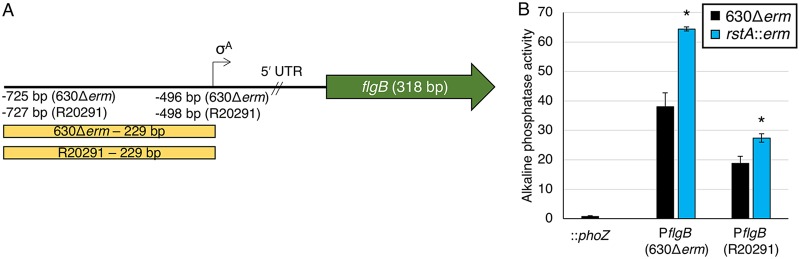
RstA represses expression of *flgB* reporter fusions. (A) A schematic of the *flgB* promoter regions for C. difficile 630 and R20291 strains. The transcriptional start site for the σ^A^-dependent promoter for strain 630 lies −496 bp upstream from the *flgB* translational start, while the R20291 strain initiates transcription −498 bp upstream (39, 56). (B) Alkaline phosphatase (AP) activity of the promoterless::*phoZ* vector in 630Δ*erm* (MC1106) and P*flgB*_630Δ_*_erm_*::*phoZ* (MC1294/MC1295) and P*flgB*_R20291_::*phoZ* (MC1296/MC1297) reporter fusions in 630Δ*erm* and the *rstA*::*erm* mutant (MC391) grown in TY medium (pH 7.4) at T_3_ (three hours after the start of transition phase [OD_600_ of 1.0]). The means and standard errors of the means for three biological replicates are shown. *, *P < *0.05, using Student’s *t* test compared to the activity observed in the 630Δ*erm* parent strain for each promoter construct.

SigD, also known as FliA or σ^28^, is a sigma factor that coordinates flagellar gene expression and directly activates *tcdR* gene expression (32). The *sigD* gene is located in a large, early-stage flagellar operon that is transcribed from a σ^A^-dependent promoter located 496 bp upstream from the first gene of the *flgB* operon (39). Interestingly, the *flgB* promoter sequences from two different C. difficile strains, the historical epidemic strain, strain 630, and a current epidemic strain, strain R20291, are identical to the σ^A^ promoter sequence through the translational start site but diverge considerably upstream of this region (Fig. S4). No additional promoter elements were identified in the strain 630 or R20291 sequences upstream of the σ^A^-dependent promoter (Fig. 3A). To determine whether RstA influences *sigD* transcription through repression of P*flgB*, promoter reporter fusions representing each strain were constructed. As anticipated, activity of the strain 630Δ*erm* and R20291 P*flgB* reporters were higher in the *rstA* mutant than in the parent strain (1.7-fold and 1.5-fold, respectively; Fig. 3B), indicating that RstA represses *flgB* and consequently, *sigD* transcription.

### RstA directly binds the *rstA*, *tcdR, flgB*, *tcdA,* and *tcdB* promoters via the conserved helix-turn-helix DNA-binding domain.

To determine whether RstA directly binds target DNA, a variety of *in vitro* electrophoretic gel shift assays were attempted, but no binding was observed in any condition tested. We considered that the lack of RstA-DNA interaction by gel shift may occur because of the absence of a cofactor, such as a quorum-sensing peptide, or because of a transient complex or oligomerization state. To overcome this obstacle, we performed biotin-labeled DNA pulldown assays to assess the DNA-binding capacity of RstA under native conditions. Biotinylated DNA was coupled to streptavidin beads as bait and incubated with cell lysates expressing either full-length RstA-FLAG or RstAΔHTH-FLAG protein. Specifically bound proteins were eluted and analyzed by Western blotting using FLAG M2 antibody.

**FIG 4:**
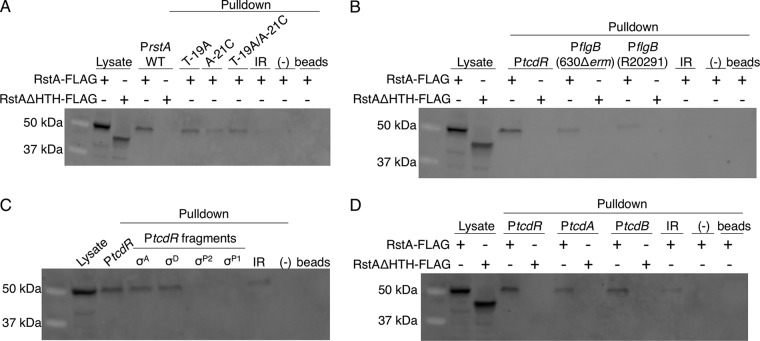
RstA binds to the *rstA*, *tcdR*, *flgB*, *tcdA*, and *tcdB* promoters. Western blot analysis using FLAG M2 antibody to detect recombinant RstA-3XFLAG or RstAΔHTH-3XFLAG in cell lysates or following biotin-labeled DNA pulldown assays. As a control, cell lysate expressing the RstA-3XFLAG construct (MC1004) or the RstAΔHTH-3XFLAG construct (MC1028) is included in the first lane or two of each Western blot shown. Additional negative controls in each panel include unbiotinylated full-length *rstA* promoter (−) and beads-only controls to ensure that RstA does not interact with the beads nonspecifically. The biotin-labeled fragments used as bait are of the 115-bp wild-type, T-19A, A-21C, or T-19A/A-21C *rstA* promoters or of the 380-bp intergenic region upstream of the *rstA* promoter (IR; see Fig. 2; present in all panels) (A), the full-length *tcdR* (446-bp) or the 630Δ*erm* or R20291 *flgB* (229-bp) promoters (B), the full-length *tcdR* (446-bp), σ^A^-dependent (92-bp), σ^D^-dependent (116-bp), σ^TcdRP2^-dependent (188-bp), or σ^TcdRP1^-dependent (112-bp) promoters (C), or the full-length *tcdR* (446-bp), *tcdA* (511-bp), or *tcdB* (501-bp) promoters (D). All promoter fragments were bound to streptavidin-coated magnetic beads and incubated with C. difficile cell lysates grown in TY medium (pH 7.4) supplemented with 2 µg/ml thiamphenicol and 1 µg/ml nisin to mid-log phase (OD_600_ of 0.5 to 0.7), expressing either the RstA-3XFLAG construct (MC1004) or the RstAΔHTH-3XFLAG construct (MC1028).

We first tested the ability of RstA to directly interact with its own promoter. RstA-FLAG protein was recovered using the wild-type *rstA* promoter region as bait, demonstrating specific interaction of the RstA protein (Fig. 4A). However, the P*rstA* fragment did not capture RstAΔHTH-FLAG protein, indicating that the conserved HTH domain of RstA is essential for DNA binding. In addition to the wild-type *rstA* promoter, the P*rstA* T-19A and P*rstA* A-21C variants that eliminated RstA-dependent regulation *in vivo* were used as bait (Fig. 1D). Both the P*rstA* T-19A and P*rstA* T-19A/A-21C variants captured significantly less RstA-FLAG than the wild-type promoter, suggesting that at least the T-19A nucleotide facilitates RstA interaction (Fig. 4A and Fig. S5A). The intergenic region upstream of the *rstA* promoter (Fig. 1A, IR) did not recover the full-length RstA-FLAG, indicating that RstA recognizes a specific DNA sequence within the promoter region. Finally, RstA-FLAG did not interact with unlabeled streptavidin beads nonspecifically (Fig. 4A and Fig. S5A). Altogether, these data demonstrate that RstA functions as a DNA-binding protein that directly and specifically binds its own promoter to repress transcription.

To determine whether RstA directly binds DNA to repress the transcription of genes encoding toxin regulators, we examined RstA binding to the *flgB* and *tcdR* promoter regions. RstA-FLAG protein bound specifically to the full-length *tcdR* promoter region, as well as the 630 and R20291 *flgB* promoters (Fig. 4B and Fig. S5B). Again, the HTH domain was required for these RstA-promoter interactions. To identify which internal promoter elements directly interact with RstA, previously characterized *tcdR* promoter fragments were used as bait (Fig. 2B), with the exception of a longer σ^A^-dependent promoter fragment (92 bp rather than 76 bp) to limit potential steric hindrance of RstA binding due to the 5ʹ biotin label. This longer 92-bp P*tcdR*(σ^A^) fragment exhibited the same RstA-dependent regulation in reporter assays as the 76-bp reporter (Fig. S6). RstA-FLAG bound to the σ^A^-dependent and σ^D^-dependent *tcdR* promoter fragments but was not recovered from either of the σ^TcdR^-dependent promoters (Fig. 4C and Fig. S5B), corroborating the reporter fusion results that demonstrated RstA repression of only the σ^A^-dependent and σ^D^-dependent *tcdR* promoter elements.

DNA pulldown assays were also performed to ascertain whether RstA directly binds to the *tcdA* and *tcdB* promoters. Both of the toxin promoters captured the full-length RstA-FLAG protein and failed to recover the RstAΔHTH-FLAG protein (Fig. 4D and Fig. S5B). These data provide direct biochemical evidence that RstA represses *flgB*, *tcdR*, *tcdA*, and *tcdB* transcription by binding to the promoter regions of these genes.

### RstA represses toxin gene expression independently of SigD-mediated toxin regulation.

**FIG 5:**
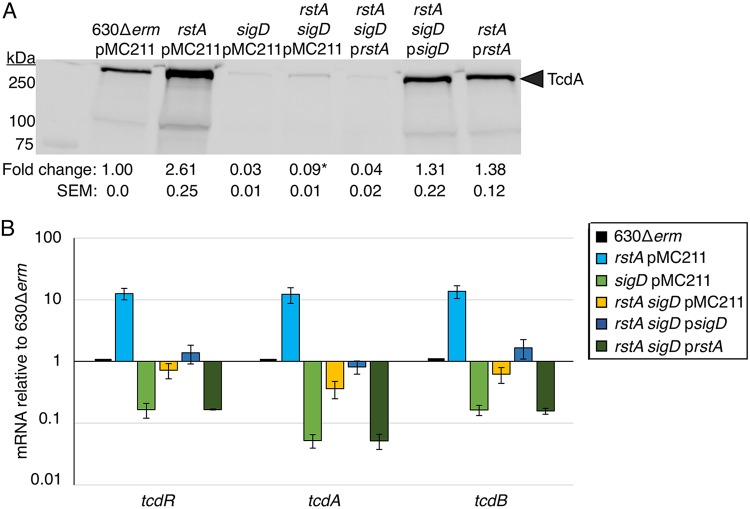
RstA represses toxin gene expression independently of SigD-mediated regulation. (A) Western blot analysis of TcdA in 630Δ*erm* pMC211 (MC282; vector control), *rstA* pMC211 (MC1224; vector control), *sigD*::*erm* pMC211 (MC506; vector control), *rstA sigD*::*erm* pMC211 (MC1281), *rstA sigD*::*erm* p*PcprA-rstA* (MC1282), *rstA sigD*::*erm* p*PcprA-sigD* (MC1283), and *rstA* pP*cprA-rstA* (MC1225) grown in TY medium (pH 7.4) supplemented with 2 µg/ml thiamphenicol and 1 µg/ml nisin, at 24 h. The corresponding image showing total protein is shown in Fig. S8A in the supplemental material. (B) qRT-PCR analysis of *tcdR*, *tcdA*, and *tcdB* transcript levels in 630Δ*erm* pMC211 (MC282; vector control), *rstA* pMC211 (MC1224; vector control), *sigD*::*erm* pMC211 (MC506; vector control), *rstA sigD*::*erm* pMC211 (MC1281), *rstA sigD*::*erm* p*PcprA-rstA* (MC1282), and *rstA sigD*::*erm* p*PcprA-sigD* (MC1283) grown in TY medium (pH 7.4) supplemented with 2 µg/ml thiamphenicol and 1 µg/ml nisin, at T_3_ (three hours after the entry into stationary phase). The means and standard errors of the means for three biological replicates are shown. *, *P < *0.05 by Student’s *t* test between *sigD::erm* pMC211 and *rstA sigD*::*erm* pMC211.

Our data indicate that RstA represses toxin gene expression directly by binding to the *tcdA* and *tcdB* promoter regions and indirectly by repressing transcription of the sigma factors *tcdR* and *sigD*, which activate toxin gene expression. The biotin pulldown data suggest that RstA represses toxin gene expression through a multitiered regulatory pathway. To test whether direct repression of *tcdA* and *tcdB* transcription by RstA is physiologically relevant and independent of SigD, we created an *rstA sigD* double mutant and examined the impact of each mutation on toxin production. To aid in construction of an *rstA sigD* double mutant, we utilized the recently developed CRISPR-Cas9 system modified for use in C. difficile to create an unmarked, nonpolar deletion of *rstA* in the 630Δ*erm* and *sigD*::*erm* backgrounds (Fig. S7) (40). TcdA protein levels were ∼3-fold higher in the *rstA sigD* double mutant than in the *sigD* mutant (Fig. 5A; total protein loaded shown in Fig. S8A), indicating that RstA represses toxin production independently of SigD. Overexpression of *rstA* in the *rstA sigD* mutant returned TcdA protein to the levels found in the *sigD* mutant. Likewise, a previously characterized *sigD* overexpression construct (11, 41) restored TcdA to wild-type levels in the *rstA sigD* mutant, further supporting that SigD and RstA regulate toxin production independently (Fig. 5A). In addition, transcript levels of *tcdA*, *tcdB,* and *tcdR* were increased in the *rstA sigD* mutant compared to the levels in the *sigD* mutant (Fig. 5B), mirroring the TcdA protein results. Altogether, these data provide further evidence that RstA is major regulator of toxin production that directly and indirectly represses toxin gene expression independently of SigD.

### RstA DNA-binding activity requires the species-specific C-terminal domains.

**FIG 6:**
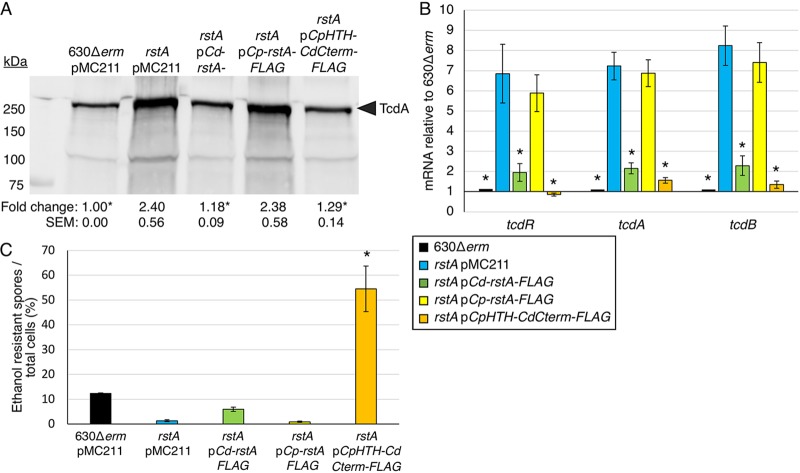
A hybrid *rstA* construct containing the C. perfringens DNA-binding domain with the C. difficile Spo0F-like and quorum-sensing-like domains complements C. difficile *rstA* toxin production and sporulation. (A) Western blot analysis of TcdA in 630Δ*erm* pMC211 (MC282; vector control), *rstA*::*erm* pMC211 (MC505; vector control), *rstA*::*erm* pP*cprA-rstA3XFLAG* (MC1004), *rstA*::*erm* pP*cprA-Cp-rstA3XFLAG* (MC1324), and *rstA*::*erm* pP*cprA-CpHTHCdCterminal3XFLAG* (MC1257) grown in TY medium, pH 7.4, supplemented with 2 µg/ml thiamphenicol and 1 µg/ml nisin, at H_24_. The corresponding image showing total protein is shown in Fig. S8B. (B) qRT-PCR analysis of *tcdR*, *tcdA*, and *tcdB* transcript levels in 630Δ*erm* pMC211 (MC282; vector control), *rstA::erm* pMC211 (MC505; vector control), *rstA*::*erm* pP*cprA-rstA3XFLAG* (MC1004), *rstA*::*erm* pP*cprA-Cp-rstA3XFLAG* (MC1324), and *rstA*::*erm* pP*cprA-CpHTHCdCterminal3XFLAG* (MC1257) grown in TY medium, pH 7.4, supplemented with 2 µg/ml thiamphenicol and 1 µg/ml nisin, at T_3_ (three hours after the entry into stationary phase). (C) Ethanol-resistant spore formation of 630Δ*erm* pMC211 (MC282; vector control), *rstA*::*erm* pMC211 (MC505; vector control), *rstA*::*erm* pP*cprA-rstA3XFLAG* (MC1004), *rstA*::*erm* pP*cprA-Cp-rstA3XFLAG* (MC1324), and *rstA*::*erm* pP*cprA-CpHTHCdCterminal3XFLAG* (MC1257) grown on 70:30 sporulation agar supplemented with 2 µg/ml thiamphenicol and 1 µg/ml nisin. Sporulation frequency is calculated as the number of ethanol-resistant spores divided by the total number of cells enumerated at H_24_ as detailed in Materials and Methods. The means and standard errors of the means for at least three independent biological replicates are shown; asterisks represent *P ≤ *0.05 by one-way ANOVA, followed by Dunnett’s multiple-comparison test compared to *rstA* pMC211 (MC505).

The observation that RstA does not bind to target DNA in the tested *in vitro* conditions but does bind DNA in cell lysates suggests that a cofactor is required for RstA DNA-binding activity. We hypothesize that a small, quorum-sensing peptide serves as an activator for RstA DNA binding, as has been observed for other members of the RRNPP family (23–25, 42–44). To test this, we expressed RstA orthologs of other clostridial species (Fig. S9A) (45), including Clostridium acetobutylicum, Clostridium perfringens, and *Clostridium* (*Paeniclostridium*) *sordellii* in the C. difficile *rstA* mutant background. Only the C. perfringens RstA was stably produced in C. difficile (Fig. S9B). However, expression of the C. perfringens *rstA* ortholog failed to restore TcdA protein to wild-type levels (Fig. 6A; total protein loaded shown in Fig. S8B). C. perfringens RstA may be unable to repress C. difficile toxin production because the C. perfringens DNA-binding domain cannot recognize the C. difficile DNA target sequences and/or because the DNA-binding activity of C. perfringens RstA is not functional in C. difficile. To distinguish between these possibilities, we constructed a chimeric protein containing the C. perfringens DNA-binding domain (M1-Y51) fused to the C-terminal domains of the C. difficile RstA protein (herein known as *Cp*HTH-*Cd*CterminalFLAG) and examined the function of this chimeric RstA in the C. difficile *rstA* mutant. The RstA chimera restored C. difficile TcdA levels to those observed in the parent strain (Fig. 6A), indicating that the C. perfringens DNA-binding domain is functional in C. difficile. To confirm these results, we performed qRT-PCR analyses of *tcdR*, *tcdA*, and *tcdB* genes in these strains. The full-length C. perfringens RstA did not complement toxin gene expression in the C. difficile *rstA* mutant, while the *Cp*HTH-*Cd*Cterminal-FLAG chimeric RstA restored toxin gene transcript levels back to those observed in the parent strain (Fig. 6B), corroborating our previous results. These data strongly suggest that the C-terminal portion of RstA responds to species-specific signals to control the N-terminal DNA-binding activity.

Finally, we assessed the ability of a C. perfringens RstA to complement the low sporulation frequency of the C. difficile *rstA* mutant. Overexpressing the full-length C. perfringens RstA did not complement sporulation in the C. difficile *rstA* mutant (Fig. 6C). Unexpectedly, a hypersporulation phenotype was observed when the *Cp*HTH-*Cd*C-terminalFLAG RstA chimera was expressed in the *rstA* mutant (Fig. 6C), indicating that the chimeric C. perfringens-C. difficile RstA promotes C. difficile sporulation to even higher levels than the native C. difficile RstA does. This hypersporulation phenotype suggests that the C. perfringens HTH portion of the chimeric RstA protein alters the structure or activity of RstA to increase the positive effect on early sporulation events. These data warrant further investigation into the molecular mechanisms by which the C-terminal domains of RstA cooperate with the DNA-binding domain to promote sporulation.

## DISCUSSION

The production of exotoxins and the ability to form quiescent endospores are two essential features of C. difficile pathogenesis. The regulatory links between toxin production and spore formation are complex and poorly understood. Some conserved sporulation regulatory factors, including Spo0A, CodY, and CcpA, strongly influence toxin production, yet some of these regulatory effects appear to be dependent on the strain or are indirect (8, 36, 46–48). Further, additional environmental conditions and metabolic signals, such as temperature and proline, glycine, and cysteine availability (5, 6, 10, 49), impact toxin production independently of these regulators, revealing the possibility that additional unknown factors are directly involved in toxin regulation (13). The recently discovered RRNPP regulator, RstA, represses toxin production and promotes spore formation, potentially providing a direct and inverse link between C. difficile spore formation and toxin biogenesis (14).

**FIG 7:**
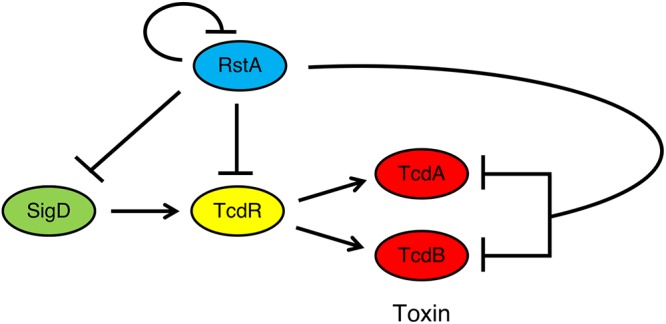
Model of RstA-mediated repression of C. difficile toxin production. SigD, the flagellum-specific sigma factor, directly induces gene transcription of *tcdR*, the toxin-specific sigma factor. Toxin gene expression is then directed by TcdR. RstA inhibits production of TcdA and TcdB and its own gene expression by directly binding to and repressing transcription of *sigD*, *tcdR*, *tcdA*, *tcdB*, and *rstA,* creating a complex, multitiered regulatory network to ensure that the toxin gene expression is appropriately timed in response to the signal(s) that activate RstA.

In this study, we show that RstA is a major, direct transcriptional regulator of C. difficile toxin gene expression. RstA inhibits toxin production by directly binding to the *tcdA* and *tcdB* promoters and repressing their transcription. RstA reinforces this repression by directly downregulating gene expression of *tcdR*, which encodes the sole sigma factor that drives *tcdA* and *tcdB* transcription. Finally, RstA directly represses the *flgB* promoter, inhibiting gene expression of the flagellum-specific sigma factor, SigD. SigD activates motility gene transcription but is also required for full expression of *tcdR* (11, 12). RstA repression of each major component in the toxin regulatory pathway creates a multitiered network in which RstA directly and indirectly controls *tcdA* and *tcdB* gene expression (Fig. 7).

RstA is the third characterized transcriptional repressor that directly binds to promoter regions for *tcdR*, *tcdA,* and *tcdB*, following two other transcriptional repressors, CodY and CcpA (8, 9, 36), The *in vivo* contribution of this reinforced repression of *tcdA* and *tcdB* transcription by CodY, CcpA, and RstA remains unknown. Interestingly, recent evidence has demonstrated that *tcdR* gene expression serves as a bistable switch that determines whether individual C. difficile cells within a population produce TcdA and TcdB, creating a divided population of toxin-OFF and toxin-ON cells (50). TcdR governs this bistability state by maintaining low basal expression levels, allowing for small changes to result in stochastic gene expression, and by positively regulating its own expression, establishing a positive-feedback loop that bolsters the toxin-ON state (50). CodY was found to influence the population so that fewer cells produced toxin, but CcpA and RstA were not tested (50). We predict that both CcpA and RstA would bias the population of cells to a toxin-OFF state. Altogether, the tight control of *tcdR* transcription, reinforced by direct repression of *tcdA* and *tcdB* transcription by CcpA, CodY, and RstA, results in the convergence of multiple regulatory pathways at the bistable *tcdR* promoter to coordinate toxin production in response to nutritional and species-specific signals. This complex regulation ensures that the energy-intensive process of toxin production is initiated only to benefit the bacterium under the appropriate conditions.

Importantly, RstA is the first transcriptional regulator demonstrated to directly control *flgB* transcription initiation. To date, none of the previously identified regulators of *flgB* expression, including Spo0A, SigH, Agr, Hfq, SinR, and SinR′, have been shown to bind promoter DNA and regulate flagellar gene expression through transcription initiation (46, 51–54). *flgB* expression is further regulated posttranscriptionally via a c-di-GMP riboswitch and a flagellar switch, both of which are located within the large, 496-bp 5′ untranslated region (39, 55, 56); however, the impact of RstA-mediated repression of *flgB* gene expression through additional pathways has not yet been explored.

Although we have identified several direct RstA targets, the sequence required to recruit RstA to target promoters remains unclear. The *rstA* promoter contains a near-perfect inverted repeat; however, this sequence is AT rich, as is the case for many C. difficile promoters. Imperfect inverted repeats were also found overlapping the −35 consensus sequences of the *tcdA*, *tcdB*, *flgB*, and σ^A^-dependent *tcdR* promoters, and immediately upstream of the σ^D^-dependent *tcdR* promoter (Fig. S10) (57), suggesting that RstA inhibits transcription at these promoters by sterically obstructing RNA polymerase docking. No clear consensus sequence defining an RstA box is delineated from these sequences. Other RRNPP regulators have also been found to bind imperfect, palindromic repeats or specific, conserved sequences in target promoters, but to our knowledge, only PlcR has a defined binding motif (24, 58, 59). Exhaustive attempts at ChIP-seq analysis to identify the C. difficile RstA regulon proved unsuccessful; however, our data imply that RstA is a transcriptional repressor that directly controls multiple C. difficile phenotypes, and additional targets within the C. difficile genome seem likely.

The inability to recapitulate RstA-DNA binding with purified RstA *in vitro* together with the functional analysis of full-length and chimeric C. difficile and C. perfringens proteins suggest that (i) RstA DNA-binding activity requires a cofactor and (ii) this cofactor is species specific. Most RRNPP members are cotranscribed with their cognate quorum-sensing peptide precursor (19), but there are notable exceptions, including those encoded by unlinked genes (42, 60) and orphan receptors whose cognate ligands have not yet been discovered (61–63). RstA falls into this latter category, as there are no open reading frames adjacent to *rstA* that encode an apparent quorum-sensing peptide precursor. Importantly, no type of ligand other than small, quorum-sensing peptides has been identified for RRNPP proteins. In addition to RstA, other quorum-sensing factors have been implicated in C. difficile toxin production. The incomplete Agr1 and conserved Agr2 quorum-sensing systems induce toxin production through the production of a cyclic autoinducer peptide (AIP) that is sensed extracellularly (52, 64, 65); however, it is highly unlikely that the extracellular AIP molecule directly interacts with the cytosolic RstA protein. In addition, the interspecies LuxS-derived autoinducer-2 (AI-2) quorum-sensing molecule was found to increase C. difficile *tcdA* and *tcdB* gene expression, but not *tcdR* gene expression (66), indicating that AI-2 does not signal through RstA either. Identification of the cofactor that controls RstA activity is a high priority, as this will likely provide insight into the physiological conditions and/or metabolites that influence C. difficile TcdA and TcdB production.

Finally, as RstA is necessary for efficient C. difficile spore formation, the possibility remains that species-specific signaling is required for RstA-dependent control of early sporulation and that RstA coordinates C. difficile toxin production and spore formation in response to the same signal(s). Elucidating the molecular mechanisms that govern RstA activity will provide important insights into the regulatory control between sporulation and toxin production, reveal host cues and conditions that lead to increased toxin production, and help delineate the early sporulation events that control C. difficile Spo0A phosphorylation and activation.

## MATERIALS AND METHODS

### Bacterial strains and growth conditions.

The bacterial strains and plasmids used in the study are listed in Table 1. Clostridioides difficile strains were routinely cultured in BHIS or TY medium (pH 7.4) supplemented with 2 to 5 µg/ml thiamphenicol and/or 1 µg/ml nisin throughout growth as needed (67). Overnight cultures of C. difficile were supplemented with 0.1% taurocholate and 0.2% fructose to promote spore germination and prevent sporulation, respectively, as indicated (67, 68). C. difficile strains were cultured in a 37°C anaerobic chamber with an atmosphere of 10% H_2_, 5% CO_2_, and 85% N_2_, as previously described (69). Escherichia coli strains were grown at 37°C in LB (70) with 100 µg/ml ampicillin and/or 20 µg/ml chloramphenicol as needed. Kanamycin (50 µg/ml) was used for counterselection against E. coli HB101 pRK24 after conjugation with C. difficile, as previously described (71).

**TABLE 1:** Bacterial strains and plasmids used in this study

Plasmid or strain	Relevant genotype or feature(s)	Source, construction, or reference
Plasmids		
pRK24	Tra^+^ Mob^+^ *bla, tet*	78
pJK02	E. coli-C. difficile shuttle vector; *catP*, *cas9*, *pyrE* sgRNA, *pyrE* homology region	40
pMC123	E. coli-C. difficile shuttle vector; *bla catP*	29
pMC211	pMC123 P*cprA*	77
pMC358	pMC123 ::*phoZ*	75
pMC367	pMC123 P*cprA*-*rstA* (*CD3668*)	14
pMC533	pMC123 P*cprA*-*rstA* (*C. sordellii* ATCC 9714)	This study
pMC543	pMC123 P*rstA*_489_::*phoZ*	14
pMC559	pMC123 P*rstA*_A-27T_::*phoZ*	This study
pMC560	pMC123 P*rstA*_T-23A_::*phoZ*	This study
pMC561	pMC123 P*rstA*_T-19A_::*phoZ*	This study
pMC562	pMC123 P*rstA*_A-18T_::*phoZ*	This study
pMC563	pMC123 P*rstA*_T-17A_::*phoZ*	This study
pMC573	pMC123 P*rstA*_A-27C_::*phoZ*	This study
pMC574	pMC123 P*rstA*_A-24G_::*phoZ*	This study
pMC575	pMC123 P*rstA*_A-21C_::*phoZ*	This study
pMC576	pMC123 P*rstA*_T-19G_::*phoZ*	This study
pMC660	pMC123 P*rstA*_115_::*phoZ*	This study
pMC675	pMC123 P*cprA*-*rstA*-3XFLAG	This study
pMC676	pMC123 P*rstA*_IR_(380 bp)::*phoZ*	This study
pMC677	pMC123 P*rstA*_231_::*phoZ*	This study
pMC678	pMC123 P*rstA*_291_::*phoZ*	This study
pMC682	pMC123 P*cprA-rstA*ΔHTH-3XFLAG	This study
pMC713	pMC123 P*tcdR*::*phoZ*	This study
pMC726	pJK02 with *rstA* homology region	This study
pMC729	pMC726 with *rstA* sgRNA (oMC1724)	This study
pMC752	pMC123 P*tcdR*(σ^A^-92 bp)::*phoZ*	This study
pMC753	pMC123 P*tcdR*(σ^D^)::*phoZ*	This study
pMC754	pMC123 P*tcdR*(P2 σ^TcdR^)::*phoZ*	This study
pMC755	pMC123 P*tcdR*(P1 σ^TcdR^)::*phoZ*	This study
pMC780	pMC123 P*cprA-rstA* (C. perfringens S13)	This study
pMC787	pMC123 P*cprA-rstA* (C. acetobutylicum ATCC 824)	This study
pMC795	pMC123 P*tcdA*::*phoZ*	This study
pMC796	pMC123 P*tcdB*::*phoZ*	This study
pMC798	pMC123 P*cprA*-*rstACpHTHCdCterminal-*3XFLAG	This study
pMC812	pMC123 P*tcdR*(σ^A^-76 bp)::*phoZ*	This study
pMC817	pRT1824 P*flgB* (630)::*phoZ*	This study
pMC818	pRT1824 P*flgB* (R20291)::*phoZ*	This study
pMC828	pMC123 P*cprA*-*rstA-*3XFLAG (C. acetobutylicum ATCC 824)	This study
pMC829	pMC123 P*cprA*-*rstA-*3XFLAG (C. perfringens S13)	This study
pMC830	pMC123 P*cprA*-*rstA-*3XFLAG (*C. sordellii* ATCC 9714)	This study
pMC888	pMC123 P*rstA*::*phoZ* P*cprA-rstA-*3XFLAG	This study
pMC889	pMC123 P*rstA*_T-19A/A-21C_::*phoZ*	This study
pRPF144	pMLT960 P*cwp2*-*gusA*	79
pRT1824	pMLT960 ::*phoZ*	This study
pSigD	pMC123 P*cprA*-*sigD*	11

E. coli strains		
HB101 pRK24	F^−^ *mcrB mrr hsdS20*(r_B_^−^ m_B_^−^) *recA13 leuB6 ara-14 proA2 lacY1 galK2 xyl-5 mtl-1 rpsL20* pRK24	B. Dupuy

C. difficile strains		
630Δ*erm*	Erm^s^ derivative of strain 630	Nigel Minton; 80
MC282	630Δ*erm* pMC211	77
MC310	630Δ*erm spo0A::erm*	77
MC391	630Δ*erm rstA::erm*	14
MC448	630Δ*erm* pMC358	75
MC480	630Δ*erm rstA::erm* pMC367	14
MC505	630Δ*erm rstA::erm* pMC211	14
MC506	630Δ*erm sigD*::*erm* pMC211	This study
MC762	630Δ*erm rstA::erm* pMC533	This study
MC773	630Δ*erm* pMC543	14
MC774	630Δ*erm rstA::erm* pMC543	14
MC830	630Δ*erm* pMC559	This study
MC831	630Δ*erm rstA::erm* pMC559	This study
MC832	630Δ*erm* pMC560	This study
MC833	630Δ*erm rstA::erm* pMC560	This study
MC834	630Δ*erm* pMC561	This study
MC835	630Δ*erm rstA::erm* pMC561	This study
MC836	630Δ*erm* pMC562	This study
MC837	630Δ*erm rstA::erm* pMC562	This study
MC838	630Δ*erm* pMC563	This study
MC839	630Δ*erm rstA::erm* pMC563	This study
MC856	630Δ*erm* pMC573	This study
MC857	630Δ*erm rstA::erm* pMC573	This study
MC858	630Δ*erm* pMC574	This study
MC859	630Δ*erm rstA::erm* pMC574	This study
MC860	630Δ*erm* pMC575	This study
MC861	630Δ*erm rstA::erm* pMC575	This study
MC862	630Δ*erm* pMC576	This study
MC863	630Δ*erm rstA::erm* pMC576	This study
MC979	630Δ*erm* pMC660	This study
MC980	630Δ*erm rstA::erm* pMC660	This study
MC1004	630Δ*erm rstA::erm* pMC675	This study
MC1008	630Δ*erm* pMC676	This study
MC1009	630Δ*erm rstA::erm* pMC676	This study
MC1010	630Δ*erm* pMC677	This study
MC1011	630Δ*erm rstA::erm* pMC677	This study
MC1012	630Δ*erm* pMC678	This study
MC1013	630Δ*erm rstA::erm* pMC678	This study
MC1028	630Δ*erm rstA::erm* pMC682	This study
MC1088	630Δ*erm* pMC713	This study
MC1089	630Δ*erm rstA::erm* pMC713	This study
MC1118	630Δ*erm* Δ*rstA*	This study
MC1133	630Δ*erm* pMC729	This study
MC1143	630Δ*erm* pMC752	This study
MC1144	630Δ*erm rstA::erm* pMC752	This study
MC1145	630Δ*erm* pMC753	This study
MC1146	630Δ*erm rstA::erm* pMC753	This study
MC1147	630Δ*erm* pMC754	This study
MC1148	630Δ*erm rstA::erm* pMC754	This study
MC1149	630Δ*erm* pMC755	This study
MC1150	630Δ*erm rstA::erm* pMC755	This study
MC1193	630Δ*erm sigD*::*erm* pMC729	This study
MC1224	630Δ*erm* Δ*rstA* pMC211	This study
MC1225	630Δ*erm* Δ*rstA* pMC367	This study
MC1249	630Δ*erm* pMC795	This study
MC1250	630Δ*erm rstA::erm* pMC795	This study
MC1251	630Δ*erm* pMC796	This study
MC1252	630Δ*erm rstA::erm* pMC796	This study
MC1257	630Δ*erm rstA::erm* pMC798	This study
MC1278	630Δ*erm* Δ*rstA sigD*::*erm*	This study
MC1281	630Δ*erm* Δ*rstA sigD*::*erm* pMC211	This study
MC1282	630Δ*erm* Δ*rstA sigD*::*erm* pMC367	This study
MC1283	630Δ*erm* Δ*rstA sigD*::*erm* pSigD	This study
MC1285	630Δ*erm* pMC812	This study
MC1286	630Δ*erm rstA::erm* pMC812	This study
MC1294	630Δ*erm* pMC817	This study
MC1295	630Δ*erm rstA::erm* pMC817	This study
MC1296	630Δ*erm* pMC818	This study
MC1297	630Δ*erm rstA::erm* pMC818	This study
MC1323	630Δ*erm rstA::erm* pMC828	This study
MC1324	630Δ*erm rstA::erm* pMC829	This study
MC1325	630Δ*erm rstA::erm* pMC830	This study
MC1433	630Δ*erm* pMC889	This study
MC1434	630Δ*erm rstA::erm* pMC889	This study
MC1435	630Δ*erm rstA::erm* pMC888	This study
RT1075	630Δ*erm sigD*::*erm*	81

Other strains		
ATCC 824	Clostridium acetobutylicum	ATCC
ATCC 9714	Clostridium sordellii	ATCC

### Strain and plasmid construction and accession numbers.

Oligonucleotides used in this study are listed in Table 2. Details of vector construction are described in the supplemental material (see Fig. S1 in the supplemental material**)**. C. difficile strains 630 (GenBank accession no. NC_009089.1) and R20291 (GenBank accession no. FN545816.1), Clostridium acetobutylicum ATCC 824 (GenBank accession no. NC_003030.1), Clostridium sordellii ATCC 9714 (GenBank accession no. APWR00000000), and Clostridium perfringens S13 (GenBank accession no. BA000016.3) were used as the templates for primer design and PCR amplification. The *rstA* ortholog from C. acetobutylicum was synthesized by Genscript (Piscataway, NJ). The Streptococcus pyogenes CRISPR-Cas9 system, which has been modified for use in C. difficile (40), was used to create a nonpolar deletion of the *rstA* gene. The 630Δ*erm* and RT1075 (*sigD*::*erm*) strains containing the *rstA*-targeted CRISPR-Cas9 plasmid (MC1133 and MC1193, respectively) were grown overnight in TY medium with 5 μg/ml thiamphenicol. The next morning, the cultures were backdiluted into fresh TY medium supplemented with 5 μg/ml thiamphenicol and 100 ng/ml anhydrous tetracycline for 24 h to induce expression of the CRISPR-Cas9 system. A small aliquot of this culture was streaked onto BHIS plates, and colonies were screened by PCR for the presence or absence of the *rstA* allele.

**TABLE 2:**
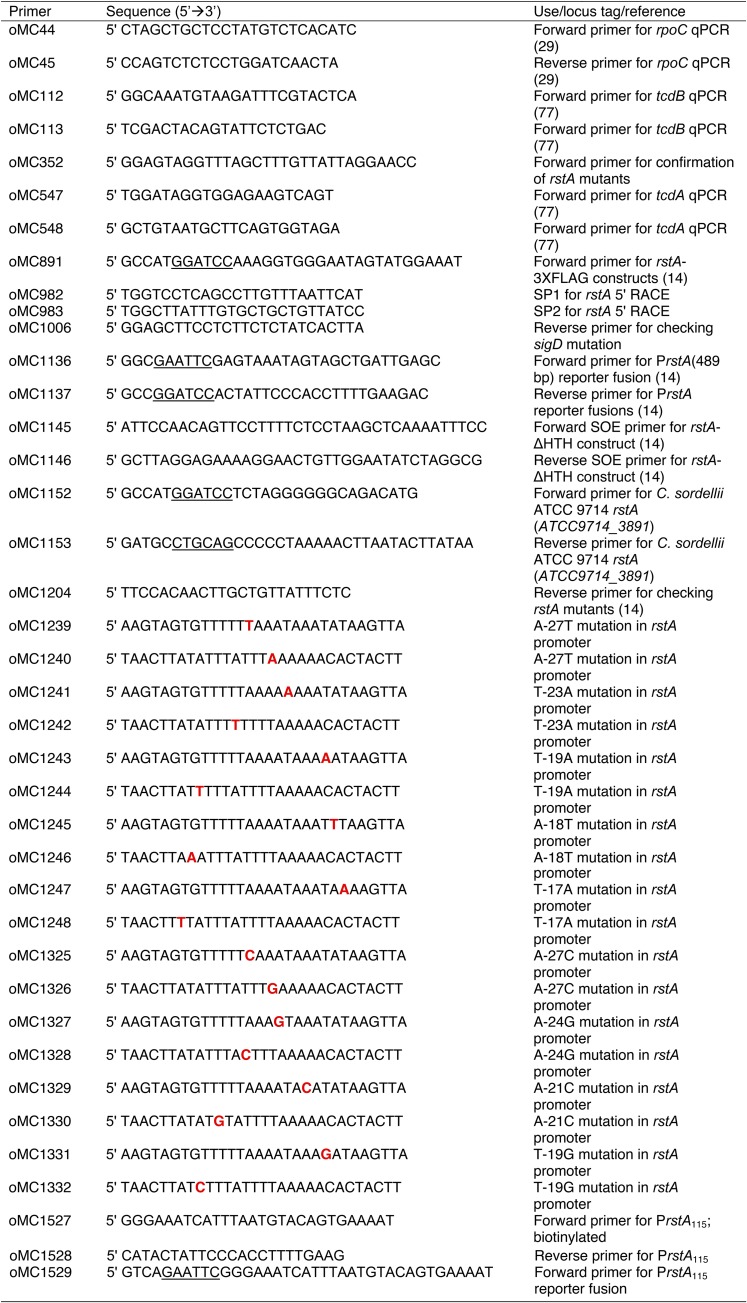

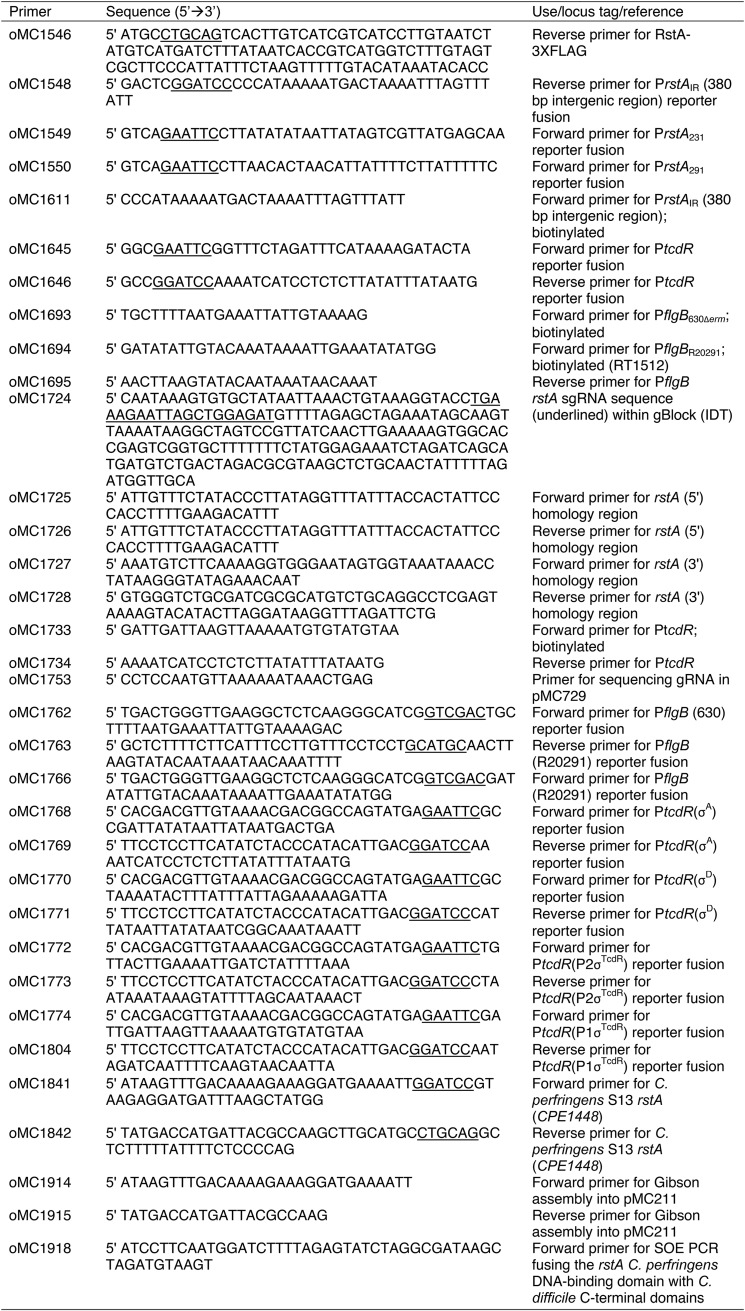

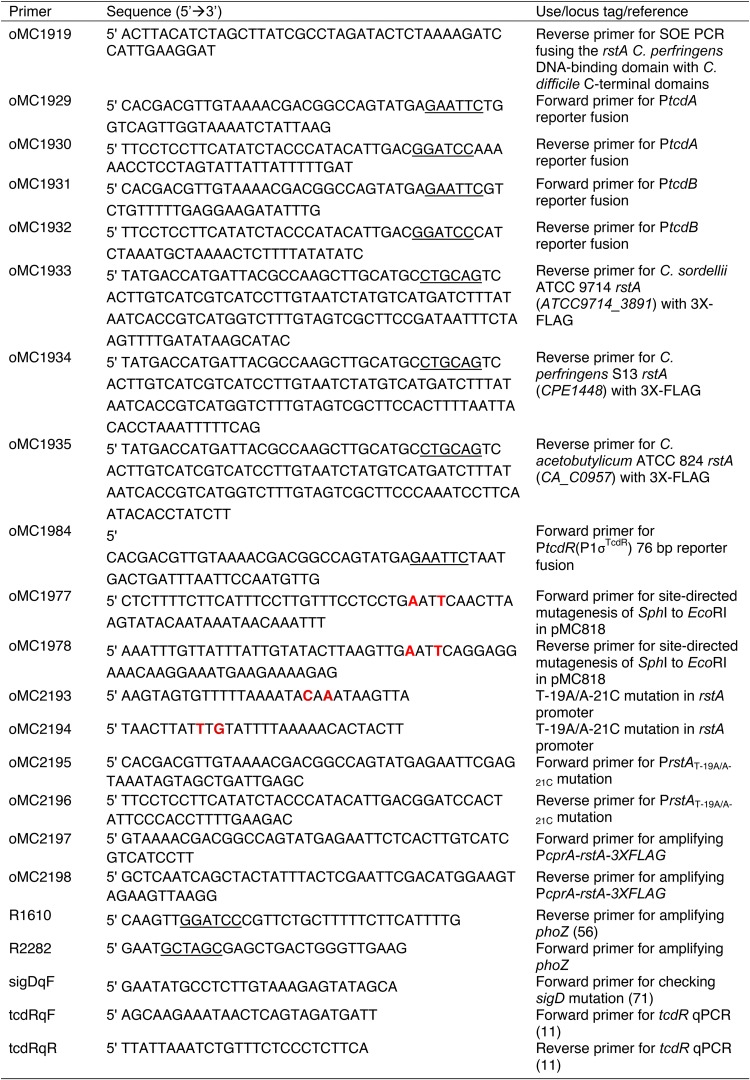
Oligonucleotides used in this study^*a*^

a Underlined nucleotides denote the restriction sites used for vector construction. Boldface red nucleotides indicate the bases mutated within the inverted repeat overlapping the *rstA* promoter.

10.1128/mBio.01991-18.1 FIG S1 DNA cloning and vector details. Download FIG S1, PDF file, 0.06 MB. Copyright © 2019 Edwards et al. 2019 Edwards et al. This content is distributed under the terms of the Creative Commons Attribution 4.0 International license.

10.1128/mBio.01991-18.2 FIG S2 The conserved helix-turn-helix (HTH) DNA-binding domain of RstA is not necessary for positively controlling sporulation but is required for the regulation of toxin production and P*rstA* expression. (A) Western blot analysis using FLAG M2 antibody in *rstA* pP*cprA-rstA-3XFLAG* (MC1004) and *rstA* pP*cprA-rstAΔHTH-3XFLAG* (MC1028) grown in TY medium, pH 7.4, at log phase (OD_600_ of 0.5). (B) Ethanol-resistant spore formation of 630Δ*erm* pMC211 (MC282; vector control), *rstA*::*erm* pMC211 (MC505; vector control), *rstA*::*erm* pP*cprA-rstA* (MC480), *rstA*::*erm* pP*cprA-rstA-3XFLAG* (MC1004), and *rstA*::*erm* pP*cprA-rstAΔHTH-3XFLAG* (MC1028) grown on 70:30 sporulation agar supplemented with 2 µg/ml thiamphenicol in the presence or absence of 1 µg/ml nisin. Sporulation frequency is calculated as the number of ethanol-resistant spores divided by the total number of cells enumerated at H_24_. The limit of detection for ethanol-resistant spores is 20 CFU/ml. Western blot analysis of TcdA (C) and the corresponding stain-free image of total protein (D) in 630Δ*erm* pMC211 (MC282; vector control), *rstA*::*erm* pMC211 (MC505; vector control), *rstA*::*erm* pP*cprA-rstA* (MC480), *rstA*::*erm* pP*cprA-rstA-3XFLAG* (MC1004), and *rstA*::*erm* pP*cprA-rstAΔHTH-3XFLAG* (MC1028) grown in TY medium, pH 7.4, at 24 h. The means and standard errors of the means for at least three independent biological replicates are shown; asterisks represent *P ≤ *0.05 by one-way ANOVA followed by Dunnett’s multiple-comparison test compared to the *rstA*::*erm* pMC211 strain (MC505). Download FIG S2, PDF file, 1.3 MB. Copyright © 2019 Edwards et al. 2019 Edwards et al. This content is distributed under the terms of the Creative Commons Attribution 4.0 International license.

10.1128/mBio.01991-18.3 FIG S3 Overexpression of RstA represses P*rstA*::*phoZ* alkaline phosphatase activity in a dose-dependent manner. Alkaline phosphatase (AP) activity of a promoterless::*phoZ* construct in the 630Δ*erm* background (MC448), the P*rstA*::*phoZ* construct expressed in 630Δ*erm* (MC773) and the *rstA*::*erm* mutant (MC774), and the P*rstA*::*phoZ* construct divergently expressed from the nisin-inducible P*cprA-rstA*-3XFLAG construct in the *rstA*::*erm* mutant (MC1435) grown on 70:30 agar supplemented with the indicated concentration of nisin at H_8_. The means and standard errors of the means for three biological replicates are shown. *, *P < *0.05 by one-way ANOVA followed by Tukey’s multiple-comparison test between the comparisons indicated in the figure. Download FIG S3, PDF file, 0.6 MB. Copyright © 2019 Edwards et al. 2019 Edwards et al. This content is distributed under the terms of the Creative Commons Attribution 4.0 International license.

10.1128/mBio.01991-18.4 FIG S4 Sequence alignment of the *flgB* promoters from *C*. *difficile* 630 and R20291 (229 bp total). The P*flgB* sequence is identical between strain 630 and its derivative 630Δ*erm*. The asterisks below the sequence indicate identical nucleotides. The −35 and −10 consensus sequence of the σ^A^-dependent promoter are marked, which are identical between both the 630 and R20291 strains (39). Download FIG S4, PDF file, 0.9 MB. Copyright © 2019 Edwards et al. 2019 Edwards et al. This content is distributed under the terms of the Creative Commons Attribution 4.0 International license.

10.1128/mBio.01991-18.5 FIG S5 Densitometry of RstA binding to promoter DNA in biotin pulldown assays. Intensity of RstA-FLAG detected by anti-FLAG Western blotting was measured using the densitometry tools provided in Image Lab (Bio-Rad). The adjusted total band volumes were normalized to RstA-FLAG recovered using either the P*rstA* promoter DNA (A) or the P*tcdR* promoter DNA (B) as bait. Representative Western blots of each biotin pulldown are shown in Fig. 4; IR is a 380-bp intergenic region upstream of the mapped *rstA* promoter that contains no promoter elements or identified RstA binding sequences (Fig. 1). The means and standard errors of the means are shown for at least three individual pulldowns for each condition. *, *P < *0.05 by one-way ANOVA followed by Dunnett’s multiple-comparison test compared to either P*rstA* or P*tcdR*. Download FIG S5, PDF file, 0.7 MB. Copyright © 2019 Edwards et al. 2019 Edwards et al. This content is distributed under the terms of the Creative Commons Attribution 4.0 International license.

10.1128/mBio.01991-18.6 FIG S6 RstA-mediated repression of two PtcdR σ^A^-dependent promoter fragments of different lengths is similar. Alkaline phosphatase (AP) activity of a promoterless::*phoZ* construct in the 630Δ*erm* background (MC448) and a longer 92-bp σ^A^-dependent P*tcdR*::*phoZ* reporter fusion in 630Δ*erm* (MC1143) and the *rstA*::*erm* mutant (MC1144) grown in TY medium, pH 7.4 at H_24_. The means and standards error of the means for four biological replicates are shown. *, *P < *0.05, using Student’s *t* test compared to the activity observed in the 630Δ*erm* parent strain for each promoter construct. The measured AP activity and calculated fold change between the *rstA* background versus the parent is the same as the 76-bp promoter region (Fig. 2B; no statistically significant difference between the 92-bp and 76-bp reporters). Download FIG S6, PDF file, 0.5 MB. Copyright © 2019 Edwards et al. 2019 Edwards et al. This content is distributed under the terms of the Creative Commons Attribution 4.0 International license.

10.1128/mBio.01991-18.7 FIG S7 PCR verification of the *rstA* and *sigD* mutations in *C*. *difficile* 630Δerm. PCR amplification from overnight cultures of 630Δ*erm*, *rstA*::*erm* (MC391), *sigD*::*erm* (RT1075), *rstA* (MC1118), and *rstA sigD*::*erm* (MC1278) strains using primers sigDqF and oMC1006 to verify the *sigD* alleles (A) (the expected sizes for the PCR products are 449 bp for the *sigD* wild-type allele and ∼2,449 bp for the *sigD::erm* insertion) and primers oMC352 and oMC1204 to verify the *rstA* alleles (B) (the expected sizes for the PCR products are 2,654 bp for the wild-type allele, ∼4,654 bp for the *rstA::erm* allele and 1,361 bp for the Δ*rstA* allele). The NEB 1-kb DNA ladder serves as the molecular marker. (C and D) A schematic representing the chromosomal organization of *sigD* (C) and *rstA* (D). The lines above the genes represent the locations of the wild-type product amplified with the indicated primers. The *rstA* gene is encoded on the complement strand. Download FIG S7, PDF file, 1.0 MB. Copyright © 2019 Edwards et al. 2019 Edwards et al. This content is distributed under the terms of the Creative Commons Attribution 4.0 International license.

10.1128/mBio.01991-18.8 FIG S8 Total protein (8 µg) transferred to nitrocellulose for TcdA Western blotting. The corresponding TGX stain-free gels used for the indicated Western blots shown in Fig. 5A (panel A above) and Fig. 6A (panel B above). For each strain tested, 8 µg of total protein was loaded onto a 4 to 15% TGX stain-free gel and imaged by a ChemiDoc (Bio-Rad) after electrophoresis. The protein was then transferred to nitrocellulose, and the Western blots were performed as described in Materials and Methods. Download FIG S8, PDF file, 1.1 MB. Copyright © 2019 Edwards et al. 2019 Edwards et al. This content is distributed under the terms of the Creative Commons Attribution 4.0 International license.

10.1128/mBio.01991-18.9 FIG S9 Alignment and stability of RstA orthologs encoded in C. difficile 630, *C. sordellii* ATCC 9714, C. perfringens S13, C. acetobutylicum ATCC 824. (A) Multiple-sequence alignment performed by EMBL-EMI Clustal Omega tool (45). The regions denoting the DNA-binding domain (from M1 to Y51), which were used in the RstA C. perfringens DNA-binding domain-C. difficile C-terminal domains hybrid construct, and the residues deleted in conserved helix-turn-helix motif for the *rstA*ΔHTH-3XFLAG construct are marked. Identical residues (*), strongly conserved residues (:), and weakly conserved residues (.) are indicated. The colors group the amino acid residues based on their physiochemical properties as follows: red, small, hydrophobic residues; blue, acidic residues; magenta, basic residues; green, hydroxyl-, sulfhydryl-, or amine-containing residue. (B) Western blot analysis using FLAG M2 antibody to detect recombinant RstA-3XFLAG proteins (∼51 kDa) expressed in 630Δ*erm* pMC211 (MC282; vector control), *rstA*::*erm* pMC211 (MC505; vector control), *rstA*::*erm* pMC828 (MC1323; Clostridium acetobutylicum *rstA* [*Ca_C0957*]*), rstA*::*erm* pMC675 (MC1004; Clostridium difficile *rstA*), *rstA*::*erm* pMC829 (MC1324; Clostridium perfringens *rstA [CPE1448*]), *rstA*::*erm* pMC830 (MC1325; *Clostridium sordelii rstA* [*ATCC9714_3891*]), and *rstA*::*erm* pMC798 (MC1257; pP*cprA*-*rstACpHTHCdCterminal-*3XFLAG) grown in TY medium supplemented with 2 µg/ml thiamphenicol and 1 µg/ml nisin at H_24_. Download FIG S9, PDF file, 2.5 MB. Copyright © 2019 Edwards et al. 2019 Edwards et al. This content is distributed under the terms of the Creative Commons Attribution 4.0 International license.

10.1128/mBio.01991-18.10 FIG S10 Alignment of inverted repeats within the promoters of direct RstA targets. The inverted repeats identified by EMBOSS Palindrome Finder (57) within each promoter that RstA directly binds are shown. The conserved −10 or −35 elements within each promoter are underlined. The predicted −35 element for PtcdR(σ^D^) begins immediately following the sequence shown here. The two nucleotides that are important for RstA binding to P*rstA* DNA are marked in red. Download FIG S10, PDF file, 0.5 MB. Copyright © 2019 Edwards et al. 2019 Edwards et al. This content is distributed under the terms of the Creative Commons Attribution 4.0 International license.

### Mapping the *rstA* transcriptional start with 5ʹ rapid amplification of cDNA ends (5ʹ RACE).

DNase I-treated RNA from the *rstA*::*erm* mutant (MC391) was obtained as described above. 5ʹ RACE was performed using the 5ʹ/3ʹ RACE kit, Second Generation (Roche), following the manufacturer’s instructions as previously reported (72). Briefly, first strand cDNA synthesis was performed using the *rstA-*specific primer oMC982, followed by purification with the High Pure PCR Product purification kit (Roche). After subsequent poly(A) tailing of first strand cDNA, PCR amplification was performed using an oligo(T) primer and the *rstA-*specific primer oMC983 with Phusion DNA Polymerase (NEB). The resulting PCR products were purified from a 0.7% agarose gel (Qiagen) and TA cloned into pCR2.1 (Invitrogen) using the manufacturer’s supplied protocols. Plasmids were isolated and sequenced (Eurofins MWG Operon) to determine the transcriptional start site (−32 bp from translational start site; *n* = 7).

### Sporulation assays.

C. difficile cultures were grown in BHIS medium supplemented with 0.1% taurocholate and 0.2% fructose until mid-exponential phase (i.e., an optical density at 600 nm [OD_600_] of 0.5), and 0.25-ml portions were spotted onto 70:30 sporulation agar supplemented with 2 µg/ml thiamphenicol and 1 µg/ml nisin as a lawn (68). After 24 h growth, ethanol resistance assays were performed as previously described (73, 74). Briefly, the cells were scraped from plates after 24 h (H_24_) and suspended in BHIS medium to an OD_600_ of 1.0. The total number of vegetative cells per milliliter was determined by immediately serially diluting and applying the resuspended cells to BHIS plates. Simultaneously, a 0.5-ml aliquot was mixed with 0.3 ml of 95% ethanol and 0.2 ml of dH_2_O to achieve a final concentration of 28.5% ethanol, vortexed, and incubated for 15 min to eliminate all vegetative cells; ethanol-treated cells were subsequently serially diluted in 1× PBS plus 0.1% taurocholate and applied to BHIS plus 0.1% taurocholate plates to determine the total number of spores. After at least 36 h of growth, CFU were enumerated, and the sporulation frequency was calculated as the total number of spores divided by the total number of viable cells (spores plus vegetative cells). A *spo0A* mutant (MC310) was used as a negative sporulation control. Statistical significance was determined using a one-way ANOVA, followed by Dunnett’s multiple-comparison test (GraphPad Prism v6.0), to compare sporulation efficiency to that of the *rstA* mutant.

### Alkaline phosphatase activity assays.

C. difficile strains containing the reporter fusions listed in Table 1 were grown and harvested on either 70:30 sporulation agar at H_8_, defined as eight hours after the cultures are applied to the plates (early stationary phase), or from TY liquid medium in stationary phase (T_3_, defined as three hours after the start of transition phase [approximately equivalent to H_8_ on plates; early stationary phase], or H_24_, defined as 24 h after the cultures are inoculated [late stationary phase]). Alkaline phosphatase assays were performed as described previously (75) with the exception that no chloroform was used for cell lysis. Technical duplicates were averaged, and the results are presented as the means and standard errors of the means for three biological replicates. The two-tailed Student’s *t* test was used to compare the activity in the *rstA* mutant to the activity in the parent strain.

### Biotin pulldown assays.

Biotin pulldown assays were performed as described by Jutras et al. (76). Briefly, a threefold excess of biotin-labeled DNA bait (30 µg) was coupled to streptavidin-coated magnetic beads (Invitrogen; binding capacity of 10 µg) in B/W buffer, and the bead-DNA complexes were washed with TE buffer to remove unbound DNA. In addition, an unbiotinylated P*rstA* (30 µg) negative control and a beads-only (dH_2_O) negative control were treated alongside the test DNA fragments to ensure that RstA did not interact nonspecifically with the streptavidin-coated magnetic beads. To determine the total amount of biotinylated-DNA bound to each bead preparation, each incubation and subsequent washes were quantitated via a Nanodrop 1000 and subtracted from the initial amount of DNA. To prepare cell lysates, C. difficile expressing either RstA-FLAG (MC1004) or RstAΔHTH-FLAG (MC1028) in the *rstA* background were grown to mid-log phase (OD_600_ of 0.5 to 0.7) in 500 ml TY medium (pH 7.4) supplemented with 2 µg/ml thiamphenicol and 1 µg/ml nisin, pelleted, rinsed with sterile water, and stored at −80°C overnight. The pellets were suspended in 4.5 ml BS/THES buffer and lysed by cycling between a dry ice/ethanol bath and a 37°C water bath. The cell lysates were vortexed for 1 min to shear genomic DNA, and cell debris was pelleted at 15K rpm for 15 min at 4°C. The supernatant, along with 10 µg salmon sperm DNA as a nonspecific competitor, was then applied to the bead-DNA complexes and rotated for 30 min at room temperature. This incubation was repeated once with additional supernatant and 10 µg salmon sperm DNA for two total incubations. The bead-DNA-protein complexes were washed seven times with BS/THES buffer supplemented with 10 µg/ml salmon sperm DNA and then without salmon sperm DNA to remove nonspecific proteins. The beads were transferred to clean microcentrifuge tubes twice during the washes to eliminate carry-over contamination. The remaining bound protein was eluted with 250 mM NaCl in Tris-HCl, pH 7.4, and the eluates were immediately analyzed by SDS-PAGE and Western blotting using FLAG M2 antibody (Sigma; see below). Each DNA bait fragment was tested in at least three independent experiments. As a control following each experiment, bait DNA was recovered by incubating the labeled beads in dH_2_O at 70°C for 10 min and analyzed on a 1.5% agarose gel to ensure that no cross-contamination occurred (data not shown). Densitometry was performed using Image Lab Software (Bio-Rad), and subsequent statistical analyses included a one-way ANOVA, followed by Dunnett’s multiple-comparison test (GraphPad Prism v6.0).

### Western blot analysis.

The indicated C. difficile strains were grown in TY medium (pH 7.4) supplemented with 2 µg/ml thiamphenicol and 1 µg/ml nisin at 37°C and harvested at H_24_ (24 h) (74). Total protein was quantitated using the Pierce Micro BCA protein assay kit (Thermo Scientific), and 8 µg of total protein was separated by electrophoresis on a precast 4 to 15% TGX stain-free gradient gel (Bio-Rad), and total protein was imaged using a ChemiDoc (Bio-Rad). Corresponding gel images for each Western blot are included in the supplemental material as indicated in the text. Protein was then transferred to a 0.45-µm nitrocellulose membrane, and Western blot analysis was conducted with either mouse anti-TcdA (Novus Biologicals) or mouse anti-FLAG (Sigma) primary antibody, followed by goat anti-mouse Alexa Fluor 488 (Life Technologies) secondary antibody. Imaging and densitometry were performed with a ChemiDoc and Image Lab software (Bio-Rad), and a one-way ANOVA, followed by Dunnett’s multiple-comparison test, was performed to assess statistical differences in TcdA protein levels between the *rstA* mutant and each *rstA* overexpression strain (GraphPad Prism v6.0). At least three biological replicates were analyzed for each strain, and a representative Western blot image is shown.

### Quantitative reverse transcription-PCR analysis.

C. difficile was cultivated in TY medium (pH 7.4) supplemented with 2 µg/ml thiamphenicol and 1 µg/ml nisin and harvested at T_3_ (defined as three hours after the start of transition phase; OD_600_ of 1.0 [approximately equivalent to H_8_ on plates]). Aliquots (3 ml) of culture were immediately mixed with 3 ml of ice-cold ethanol-acetone (1:1) and stored at −80°C. RNA was purified and DNase I treated (Ambion) as previously described (29, 35, 77), and cDNA was synthesized using random hexamers (77). Quantitative reverse transcription-PCR (qRT-PCR) analysis, using 50 ng cDNA per reaction and the SensiFAST SYBR & Fluorescein kit (Bioline), was performed in technical triplicates on a Roche Lightcycler 96. cDNA synthesis reaction mixtures containing no reverse transcriptase were included as a negative control to ensure that no genomic DNA contamination was present. Results are presented as the means and standard errors of the means for three biological replicates. Statistical significance was determined using a one-way ANOVA, followed by Dunnett’s multiple-comparison test (GraphPad Prism v6.0), to compare transcript levels between the *rstA* mutant and each *rstA* overexpression strain.
