# Supplementary material for: RstA Is a Major Regulator of Clostridioides difficile Toxin Production and Motility
Source: mBio. 2019 Mar 12;10(2):e01991-18. doi: 10.1128/mBio.01991-18 (PMC6414698; doi:10.1128/mBio.01991-18)
Supplement: FIG S1 [file mBio.01991-18-sf001.pdf]

Figure S1. DNA cloning and vector details.

pMC533: A 1.38 kb *rstA* PCR product amplified with primers oMC1152/1153 from *Clostridium sordellii* ATCC 9714 genomic DNA and was cloned into pMC211 using the *Bam*HI and *Pst*I sites.

pMC559-563; pMC573-576: Single nucleotide mutations within the 489 bp P*rstA* PCR products were generated by splicing by overlap extension PCR using primers oMC1136/1137 from two fragments generated by using primer sets oMC1239/1240 (A-27T; pMC559), oMC1241/1242 (T-23A; pMC560), oMC1243/1244 (T-19A; pMC561), oMC1245/1246 (A-18T; pMC562), oMC1247/1248 (T-17A; pMC563), oMC1325/1326 (A-27C; pMC573), oMC1327/1328 (A-24G; pMC574), oMC1329/1330 (A-21C; pMC575) and oMC1331/1332 (T-19G; pMC576). These PCR products were then cloned into pMC358 using the *Bam*HI and *Eco*RI sites to create the corresponding plasmid.

pMC660: A 115 bp P*rstA* PCR fragment amplified with primers oMC1528/1529 from *C. difficile* 630 genomic DNA was cloned into pMC358 using the *Bam*HI and *Eco*RI sites.

pMC675: A 1.43 kb *rstA*-3XFLAG PCR product amplified with primers oMC891/1546 from *C. difficile* 630 genomic DNA was cloned into pMC211 using the *Bam*HI and *Pst*I sites.

pMC676: A PCR product containing the 380 bp intergenic region located upstream from the mapped *rstA* promoter was amplified with primers oMC1136/1548 and cloned into pMC358 using the *Bam*HI and *Eco*RI sites.

pMC677: A 231 bp P*rstA* PCR product amplified with primers oMC1549/1137 from *C. difficile* 630 genomic DNA was cloned into pMC358 using the *Bam*HI and *Eco*RI sites.

pMC678: A 291 bp P*rstA* PCR product amplified with primers oMC1550/1137 from *C. difficile* 630 genomic DNA was cloned into pMC358 using the *Bam*HI and *Eco*RI sites.

pMC682: A 1.33 kb *rstA*ΔHTH-3XFLAG PCR product was generated by splicing by overlap extension PCR using primers oMC891/892/1145/1146 from *C. difficile* 630 genomic DNA and cloned into pMC211 using the *Bam*HI and *Pst*I sites.

pMC713: A 517 bp PCR fragment of the *tcdR* promoter was amplified with primers oMC1646/1647 from *C. difficile* 630 genomic DNA and cloned into pMC358 using the *Bam*HI and *Eco*RI sites.

pMC726: A 1 kb PCR product containing a 500 bp region of upstream and downstream flanking homology to *rstA* was generated by splicing by overlap PCR using PCR fragments amplified with primers oMC1726/1727/1728/1729 from *C. difficile* 630 genomic DNA and Gibson assembled into pJK02 using the *Not*I and *Xho*I sites.

pMC729: A 212 bp small guide (sg) RNA product containing a 20 bp targeting region to *rstA* (5′ TGAAAGAATTAGCTGGAGAT; identified by the CRISPRscan algorithm [Moreno-Mateos *et al.* 2015]) was synthesized by IDT (gBlock) and Gibson assembled into pMC726 using the *Kpn*I and *Mlu*I sites. The correct sgRNA sequence was verified with primer oMC1753.

pMC752: A 92 bp P*tcdR* PCR product containing the putative σ^{A}-dependent promoter amplified with primers oMC1768/1769 using *C. difficile* 630 genomic DNA and Gibson assembled into pMC358 using the *Bam*HI and *Eco*RI sites.

pMC753: A 116 bp P*tcdR* PCR product containing the putative σ^{D}-dependent promoter amplified with primers oMC1774/1775 using *C. difficile* 630 genomic DNA and Gibson assembled into pMC358 using the *Bam*HI and *Eco*RI sites.

pMC754: A 188 bp P*tcdR* PCR product containing the putative σ^{P2tcdR}-dependent promoter amplified with primers oMC1772/1773 using *C. difficile* 630 genomic DNA and Gibson assembled into pMC358 using the *Bam*HI and *Eco*RI sites.

pMC755: A 112 bp P*tcdR* PCR product containing the putative σ^{P1tcdR}-dependent promoter amplified with primers oMC1774/1804 using *C. difficile* 630 genomic DNA and Gibson assembled into pMC358 using the *Bam*HI and *Eco*RI sites.

pMC780: A 1.38 kb *rstA* PCR product was amplified using primers oMC1841/1842 from *C. perfringens* S13 genomic DNA and Gibson assembled into pMC211 using the *Bam*HI and *Pst*I sites.

pMC787: A 1.38 kb *C. acetobutylicum rstA* fragment synthesized by Genscript was Gibson assembled into pMC211 using the *Bam*HI and *Pst*I sites.

pMC795: A 511 bp PCR fragment of the *tcdA* promoter was amplified with primers oMC1929/1930 from *C. difficile* 630 genomic DNA and Gibson assembled into pMC358 using the *Bam*HI and *Eco*RI sites.

pMC796: A 501 bp PCR fragment of the *tcdB* promoter was amplified with primers oMC1931/1932 from *C. difficile* 630 genomic DNA and Gibson assembled into pMC358 using the *Bam*HI and *Eco*RI sites.

pMC798: A 1.5 kb hybrid *rstA* construct was generated by splicing by overlap PCR with PCR fragments containing the *C. perfringens* N-terminal DNA-binding domain using primers oMC1914/1919 and the *C. difficile* C-terminal protein-binding and quorum-sensing-binding domains using primers oMC1915/1918, and subsequently Gibson assembled into pMC211 using the *Bam*HI/*Pst*I sites.

pMC812: A 76 bp P*tcdR* PCR product containing the putative σ^{A}-dependent promoter amplified with primers oMC1984/1769 using *C. difficile* 630 genomic DNA and Gibson assembled into pMC358 using the *Bam*HI and *Eco*RI sites.

pMC817: A 1.76 kb P*flgB* (630)::*phoZ* insert synthesized by Genscript was amplified with primers oMC1762/2107 and Gibson assembled into pRT1824 using the *Sal*I and *Bam*HI sites.

pMC818: A 229 bp P*flgB* (R20291) PCR product amplified with primers oMC1763/1766 was Gibson assembled into pRT1824 using the *Sal*I and *Sph*I sites to create plasmid pMC749. To convert the *Sph*I site to an *Eco*RI site, site directed mutagenesis using primers oMC1977/1978, designed by the Quickchange Primer Design program (Agilent Genomics), was performed with the Stratagene Quickchange II Kit following the manufacturer's instructions.

pMC828: A 1.5 kb *rstA C. acetobutylicum* construct with a C-terminal 3XFLAG tag was amplified using primers oMC1914/1935 from pMC787 and Gibson assembled into pMC211 using the *Bam*HI and *Pst*I sites.

pMC829: A 1.5 kb *rstA C. perfringens* construct with a C-terminal 3XFLAG tag was amplified using primers oMC1914/1934 from pMC780 and Gibson assembled into pMC211 using the *Bam*HI and *Pst*I sites.

pMC830: A 1.5 kb *rstA C. sordellii* construct with a C-terminal 3XFLAG tag was amplified using primers oMC1914/1933 from pMC533 and Gibson assembled into pMC211 using the *Bam*HI and *Pst*I sites.

pMC888: A 1.5 kb *rstA C. difficile* construct with a C-terminal 3XFLAG tag was amplified using oMC2197/2198 from pMC675 and Gibson assembled into pMC543 using the *Eco*RI sites.

pMC889: The A-21C mutation was introduced into the P*rstA*$_{T\text{-}19A}$ 489 bp fragment (pMC561) via splicing by overlap extension PCR two fragments generated with primers oMC2193/2194/2195/2196) and Gibson assembled into pMC358 using the *Bam*HI and *Eco*RI sites.

pRT1824: A 1.6 bp ::*phoZ* PCR product amplified from RT1392 (*Bacillus subtilis* 49) genomic DNA (Anjuwon-Foster and Tamayo, 2017) using primers R2282 and R1610 was digested with *Nhe*I and *Bam*HI and ligated into similarly digested pRPF144 (pMLT960-based vector; Fagan and Fairweather, 2011) to create a promoterless ::*phoZ* low copy vector.

Anjuwon-Foster BR, Tamayo R. 2017. A genetic switch controls the production of flagella and toxins in *Clostridium difficile*. *PLoS Genet*. 13(3):e1006701.

Fagan RP, Fairweather N. 2011. *Clostridium difficile* has two parallel and essential Sec secretion systems. *J Biol Chem*. 286(31):27483-93.

Moreno-Mateos MA, Vejnar CE, Beaudoin J-D, Fernandez JP, Mis EK, Khokha MK, Giraldez AJ. 2015. CRISPRscan: Designing highly efficient sgRNAs for CRISPR/Cas9 targeting in vivo. *Nature Methods* 12:982-988.
