# Supplementary material for: RstA Is a Major Regulator of Clostridioides difficile Toxin Production and Motility
Source: mBio. 2019 Mar 12;10(2):e01991-18. doi: 10.1128/mBio.01991-18 (PMC6414698; doi:10.1128/mBio.01991-18)
Supplement: FIG S2 [file mBio.01991-18-sf002.pdf]

Figure S2.

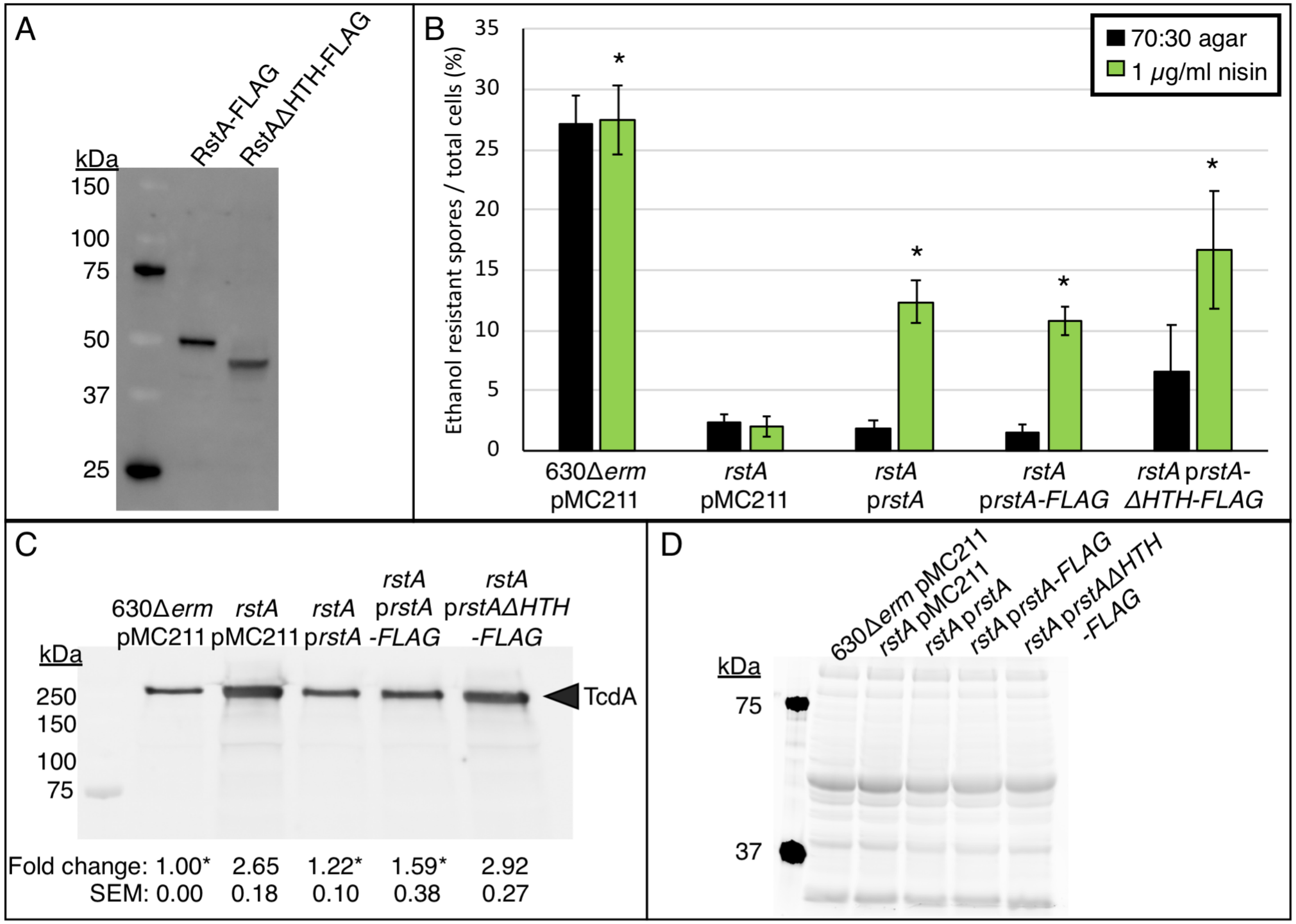

Figure S2. The conserved helix-turn-helix (HTH) DNA-binding domain of RstA is not necessary for positively controlling sporulation but is required for the regulation of toxin production and P*rstA* expression. (**A**) Western blot analysis using FLAG M2 antibody in *rstA* pP*cprA-rstA-3XFLAG* (MC1004) and *rstA* pP*cprA-rstAΔHTH-3XFLAG* (MC1028) grown in TY medium, pH 7.4, at log phase (OD_{600} = 0.5). (**B**) Ethanol resistant spore formation of 630Δ*erm* pMC211 (MC282; vector control), *rstA*::*erm* pMC211 (MC505; vector control), *rstA*::*erm* pP*cprA-rstA* (MC480), *rstA*::*erm* pP*cprA-rstA-3XFLAG* (MC1004) and *rstA*::*erm* pP*cprA-rstAΔHTH-3XFLAG* (MC1028) grown on 70:30 sporulation agar supplemented with 2 μg/ml thiamphenicol in the absence or presence of 1 μg/ml nisin. Sporulation frequency is calculated as the number of ethanol-resistant spores divided by the total number of cells enumerated at H_{24}. The limit of detection for ethanol resistant spores is 20 CFU/ml. Western blot analysis of TcdA (**C**) and corresponding Stain-free image of total protein (**D**) in 630Δ*erm* pMC211 (MC282; vector control), *rstA*::*erm* pMC211 (MC505; vector control), *rstA*::*erm* pP*cprA-rstA* (MC480), *rstA*::*erm* pP*cprA-rstA-3XFLAG* (MC1004) and *rstA*::*erm* pP*cprA-rstAΔHTH-3XFLAG* (MC1028) grown in TY medium, pH 7.4, at 24 h. The means and standard error of the means of at least three independent biological replicates are shown; asterisks represent $P \leq 0.05$ by one-way ANOVA followed by Dunnett's multiple comparison's test compared to the *rstA*::*erm* pMC211 strain (MC505).
