# Supplementary material for: RstA Is a Major Regulator of Clostridioides difficile Toxin Production and Motility
Source: mBio. 2019 Mar 12;10(2):e01991-18. doi: 10.1128/mBio.01991-18 (PMC6414698; doi:10.1128/mBio.01991-18)
Supplement: FIG S3 [file mBio.01991-18-sf003.pdf]

Figure S3.

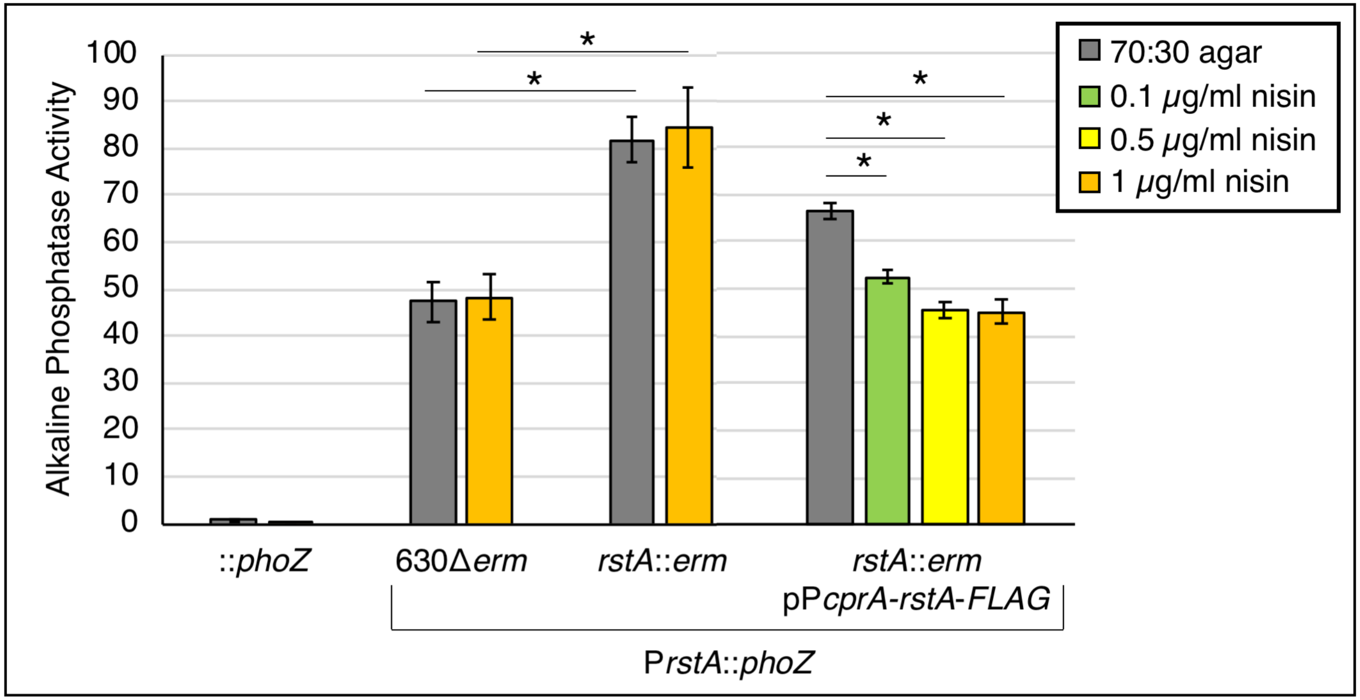

Figure S3. Overexpression of RstA represses P*rstA*::*phoZ* alkaline phosphatase activity in a dose-dependent manner. Alkaline phosphatase (AP) activity of a promoterless ::*phoZ* construct in the 630Δ*erm* background (MC448), the P*rstA*::*phoZ* construct expressed in 630Δ*erm* (MC773) and the *rstA*::*erm* mutant (MC774), and the P*rstA*::*phoZ* construct divergently expressed from the nisin-inducible P*cprA*-*rstA*-3XFLAG construct in the *rstA*::*erm* mutant (MC1435) grown on 70:30 agar supplemented with the indicated concentration of nisin at H_8. The means and standard error of the means of three biological replicates are shown. *, $P < 0.05$ by one-way ANOVA followed by Tukey's multiple comparison's test between the comparisons indicated in the figure.
