# Supplementary material for: RstA Is a Major Regulator of Clostridioides difficile Toxin Production and Motility
Source: mBio. 2019 Mar 12;10(2):e01991-18. doi: 10.1128/mBio.01991-18 (PMC6414698; doi:10.1128/mBio.01991-18)
Supplement: FIG S4 [file mBio.01991-18-sf004.pdf]

Figure S4.

```
630       ------TGCTTTTAATGAAATTATTGT-AAAAGACAAATTTAGAATAAATTTATTTTATA -176
R20291    GATATATTGTACAAATAAAATTGAAATATATGGAAGAATATAGAATAAAGATGAGATTTG -169
                *  *   *** *****    *  *  **  *** *********  *    * *

630       TTGTAAATTTTTGTAAATTTTTAATATATATGTACATCATATAAATTAAATGTTATAAAT -116
R20291    TCTAATATTTTTATAAATTAAAAATATTTTCAAAGAG--TTTATTATAAAATTGTAGACT -111
          *   * ****** ******   ***** *    * *   * **   ****  *    * *

630       ATAAATATATTAAAAATATTATAAAATTTAATATTAGAAATTAAATTAAAAAATT---AC -59
R20291    ATA--------GTGTTTATAATCTTTGTAATTATTGGTAACTAAATTTAAAAAATAAAAT -59
          ***             *** **     * * **** * ** ****** ***** *   *
                                   -35                    -10
630       ATAAAAAAAATACTTAAAAAAATATTTAAAAAATTTGTTATTTATTGTATACTTAAGTT
R20291    ATTTAATTATACACAAAAAAGATATTTAAAAAATTTGTTATTTATTGTATACTTAAGTT +1
          **  **  *      ***** **************************************
```

Figure S4. **Sequence alignment of the *flgB* promoters from *C. difficile* 630 and R20291 (229 bp total)**. The P*flgB* sequence is identical between 630 and its derivative 630Δ*erm*. The asterisks below the sequence indicate identical nucleotides. The -35 and -10 consensus sequence of the σ^A-dependent promoter are marked, which are identical between both the 630 and the R20291 strains (Soutourina, *et al*. 2013).

Soutourina OA, Monot M, Boudry P, Saujet L, Pichon C, Sismeiro O, Semenova E, Severinov K, Le Bouguenec C, Coppee JY, Dupuy B, Martin-Verstraete I. 2013. Genome-wide identification of regulatory RNAs in the human pathogen *Clostridium difficile*. *PLoS Genet* 9:e1003493.
