# Supplementary material for: RstA Is a Major Regulator of Clostridioides difficile Toxin Production and Motility
Source: mBio. 2019 Mar 12;10(2):e01991-18. doi: 10.1128/mBio.01991-18 (PMC6414698; doi:10.1128/mBio.01991-18)
Supplement: FIG S5 [file mBio.01991-18-sf005.pdf]

Figure S5.

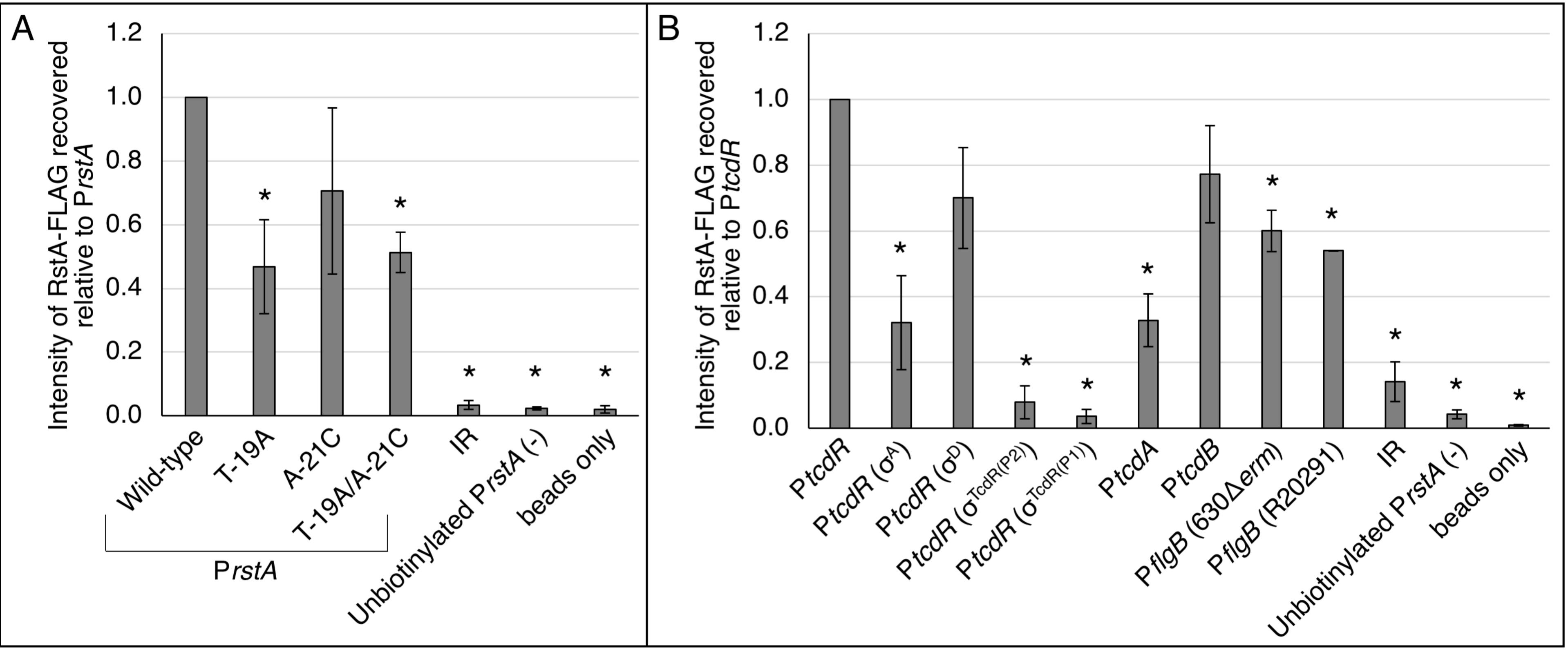

Figure S5. Densitometry of RstA binding to promoter DNA in biotin pulldown assays. Intensity of RstA-FLAG detected by anti-FLAG western blotting was measured using the densitometry tools provided in Image Lab (Bio-Rad). The adjusted total band volumes were normalized to RstA-FLAG recovered using either the P*rstA* promoter DNA (**A**) or the P*tcdR* promoter DNA (**B**) as bait. Representative western blots of each biotin pulldown are shown in Fig. 4; IR is a 380 bp intergenic region upstream of the mapped *rstA* promoter that contains no promoter elements or identified RstA binding sequences (see Fig. 1). The means and standard error of the means are shown for at least three individual pulldowns for each condition. *, $P < 0.05$ by one-way ANOVA followed by Dunnett's multiple comparison's test compared to either P*rstA* or P*tcdR*.
