# Supplementary material for: RstA Is a Major Regulator of Clostridioides difficile Toxin Production and Motility
Source: mBio. 2019 Mar 12;10(2):e01991-18. doi: 10.1128/mBio.01991-18 (PMC6414698; doi:10.1128/mBio.01991-18)
Supplement: FIG S6 [file mBio.01991-18-sf006.pdf]

Figure S6.

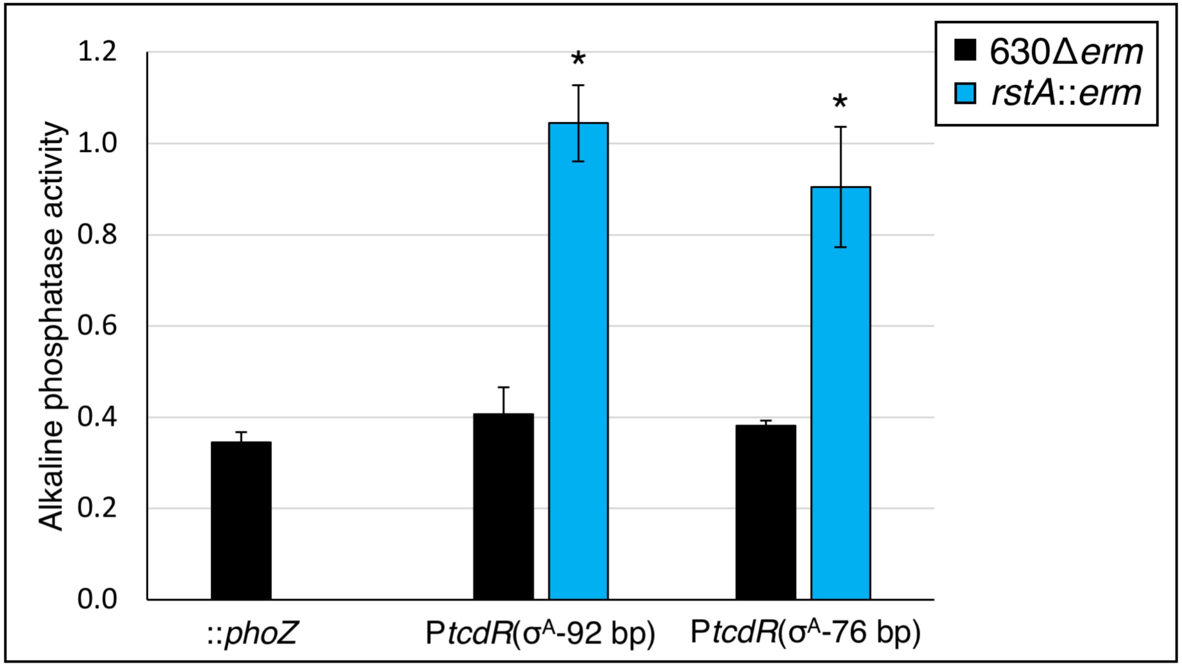

Figure S6. RstA-mediated repression of two P*tcdR* σ^A-dependent promoter fragments of different lengths is similar. Alkaline phosphatase (AP) activity of a promoterless ::*phoZ* construct in the 630Δ*erm* background (MC448) and a longer 92 bp σ^A-dependent P*tcdR*::*phoZ* reporter fusion in 630Δ*erm* (MC1143) and the *rstA*::*erm* mutant (MC1144) grown in TY medium, pH 7.4 at H_{24}. The means and standard error of the means of four biological replicates are shown. *, *P* < 0.05, using Student's *t*-test compared to the activity observed in the 630Δ*erm* parent strain for each promoter construct. The measured AP activity and calculated fold change between the *rstA* background versus the parent is the same as the 76 bp promoter region (Fig. 2B; no statistically significant difference between the 92 bp and 76 bp reporters).
