# Supplementary material for: RstA Is a Major Regulator of Clostridioides difficile Toxin Production and Motility
Source: mBio. 2019 Mar 12;10(2):e01991-18. doi: 10.1128/mBio.01991-18 (PMC6414698; doi:10.1128/mBio.01991-18)
Supplement: FIG S7 [file mBio.01991-18-sf007.pdf]

Figure S7.

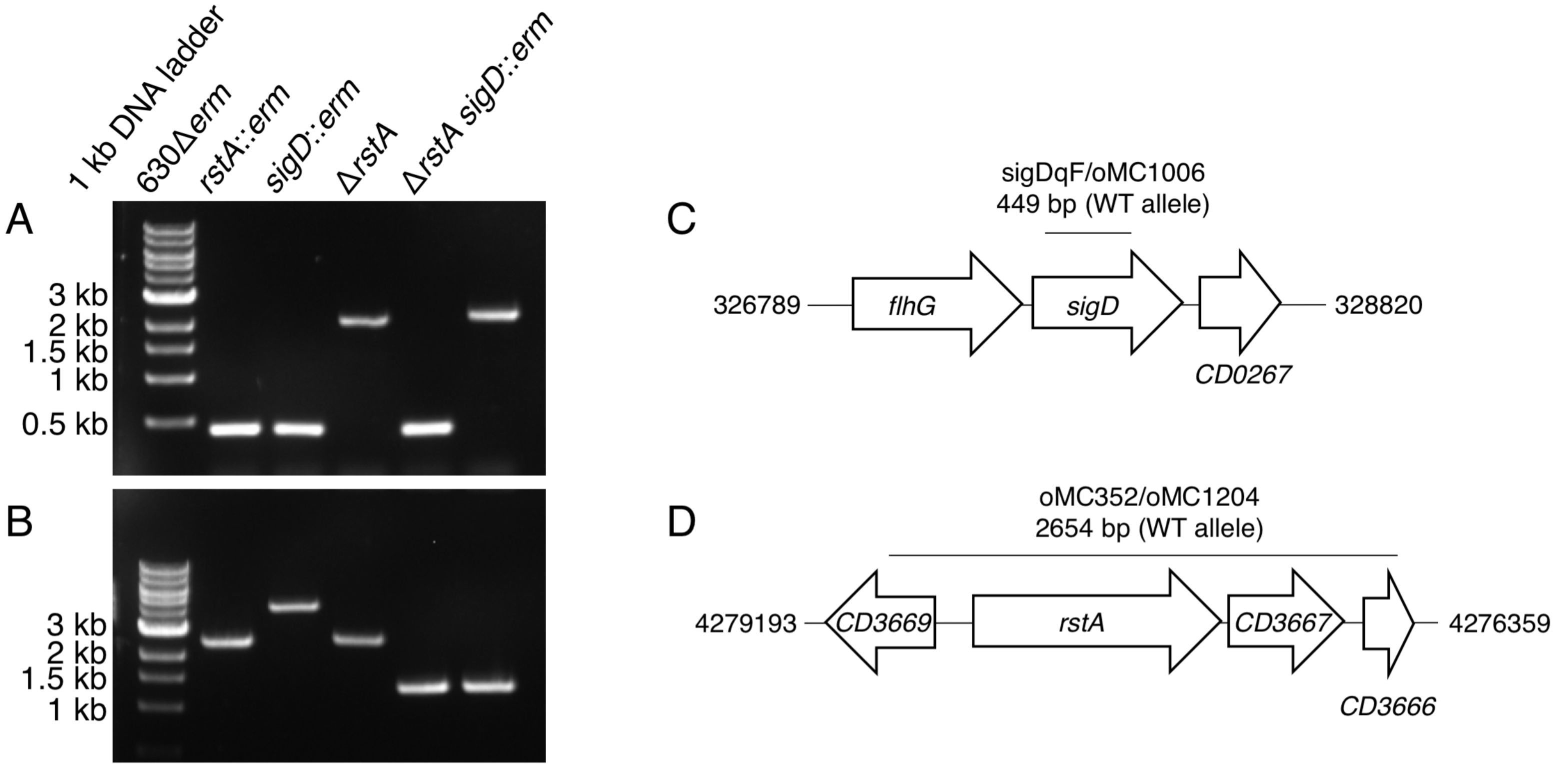

Figure S7. PCR verification of the *rstA* and *sigD* mutations in *C. difficile* 630Δ*erm*. PCR amplification from overnight cultures of 630Δ*erm*, *rstA*::*erm* (MC391), *sigD*::*erm* (RT1075), *rstA* (MC1118) and *rstA sigD*::*erm* (MC1278) strains using (**A**) primers sigDqF and oMC1006 to verify the *sigD* alleles (the expected sizes for the PCR products are 449 bp for the *sigD* wild-type allele and ~2449 bp for the *sigD::erm* insertion) and (**B**) primers oMC352 and oMC1204 to verify the *rstA* alleles (the expected sizes for the PCR products are 2654 bp for the wild-type allele, ~4654 bp for the *rstA::erm* allele and 1361 bp for the Δ*rstA* allele). The NEB 1 kb DNA ladder serves as the molecular marker. (**C**, **D**) A schematic representing the chromosomal organization of *sigD* (**C**) and *rstA* (**D**). The lines above the genes represent the location of the wild-type product amplified with the indicated primers. The *rstA* gene is encoded on the complement strand.
