# Supplementary material for: RstA Is a Major Regulator of Clostridioides difficile Toxin Production and Motility
Source: mBio. 2019 Mar 12;10(2):e01991-18. doi: 10.1128/mBio.01991-18 (PMC6414698; doi:10.1128/mBio.01991-18)
Supplement: FIG S8 [file mBio.01991-18-sf008.pdf]

Figure S8.

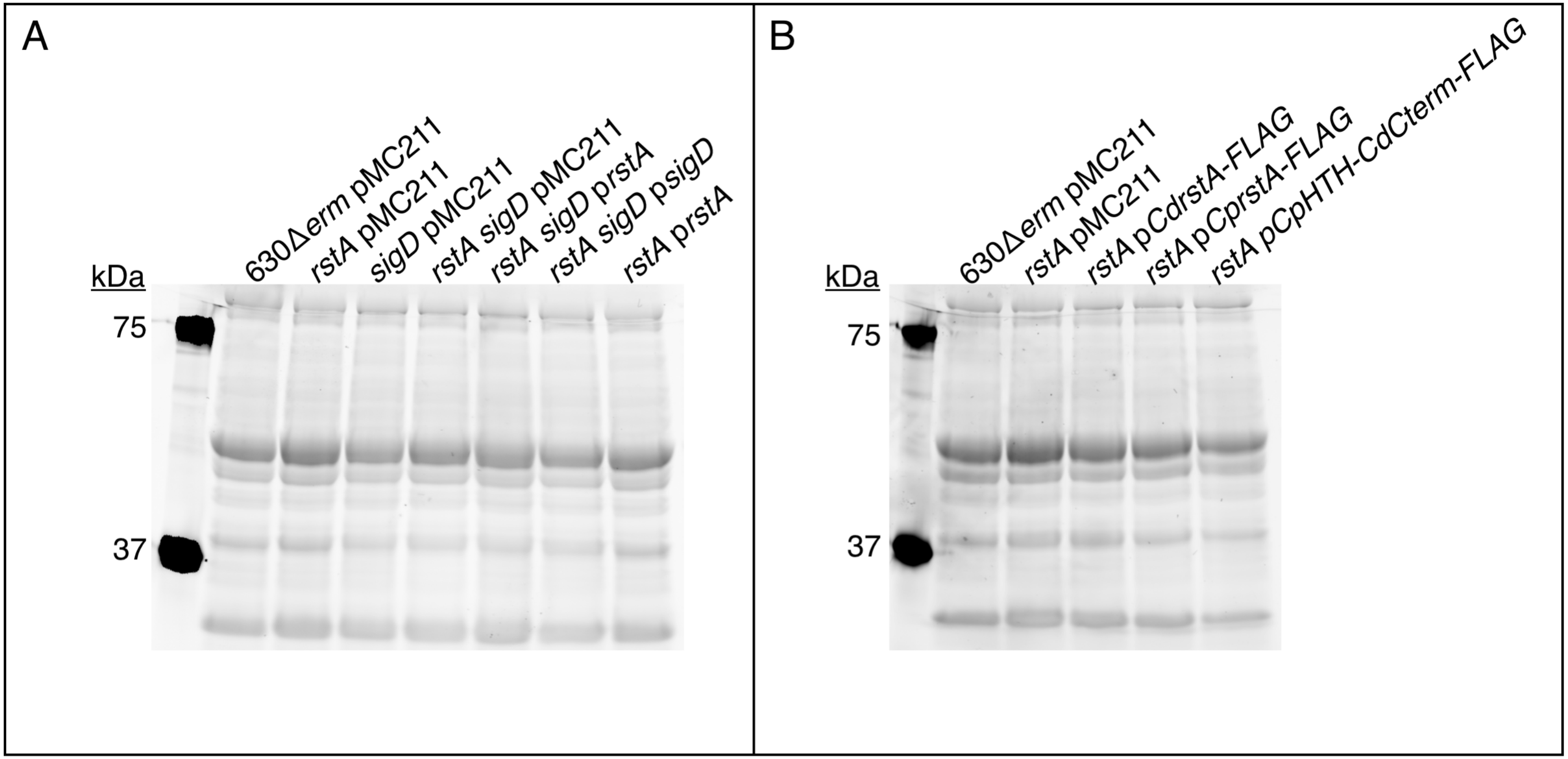


**Figure S8. Total protein (8 μg) transferred to nitrocellulose for TcdA western blotting.** The corresponding TGX Stain-free gels used for the indicated western blots shown in Fig. 5A (**Panel A above**) and Fig. 6A (**Panel B above**). For each strain tested, 8 μg of total protein was loaded onto a 4-15% TGX Stain-free gel and imaged by a ChemiDoc (Bio-Rad) after electrophoresis. The protein was then transferred to nitrocellulose and the western blots were performed as described in the Materials and Methods.
