# Supplementary material for: RstA Is a Major Regulator of Clostridioides difficile Toxin Production and Motility
Source: mBio. 2019 Mar 12;10(2):e01991-18. doi: 10.1128/mBio.01991-18 (PMC6414698; doi:10.1128/mBio.01991-18)
Supplement: FIG S9 [file mBio.01991-18-sf009.pdf]

Figure S9.

A

```
                                    DNA-binding domain (M1-Y51)
                                          ΔHTH (I10-Y46)
C. difficile RstA               MEILSLGEKIKKLRKEKNMTLKELAGDRITAAQISHIERDKSHTSYELLEYLGDKLDVSV
C. sordellii ATCC9714_31891     MEILSLGEKIKKLRKEKNLTLKELAGERITAAQISHIERDKSHTSHELLEYLASCLEVSV
C. perfringens CPE1448          MEILSLGEKIKRRRKELNMTLKELAGERITPGQISLVESGRSNPSMDLLEYIAITLNTSV
C. acetobutylicum CA_c0957      MEILSLGEKIKARRKELNMTLKDLAGDRITPGQISLVESGKSNPSMDLLEYLAGALNTSV
                                ***********  *** *:***:***:*** .*** :* .:*: * :****:.  *:.**

C. difficile RstA               EYLLETKEMQSKKITDNLILQSEIYIKENELNKAEDQIKEVLDICKRYDLMENYGRCNFL
C. sordellii ATCC9714_31891     DYLLETKEMQAKKLTDNLIIQSEIYIKCKELEKAKEEINKVMSICTEYQLIDNYGKCNLL
C. perfringens CPE1448          EYLMETEKIQAEKICLYYEQIAESAILNDDIKKGEKFIEYAIYYADKYDLIYRKAKNLAL
C. acetobutylicum CA_c0957      EYLMESEETQAEKICVYYENTAEAYMLNDELMEAEQYLEKAMYYAENYKISYRKARTLYL
                                :**:*::: *::*:        :*  :  .:: :.:. :: .:  . .*.:  . .:   *

C. difficile RstA               MANINLKKSNYNDAIENFEKALYFFIKNSDSENILKCYLNIGKIYMQEDFYKGAISHFNF
C. sordellii ATCC9714_31891     LATINLRQKDYNAAIINFEKSLYFFIQNDDKENIARCYINIGKIYMEENFHKGALSNFKF
C. perfringens CPE1448          KAEMYLKSEEYVLAQQYFLSANMLFLQINSNIDMVKTYLNIAKISLHSKAYYSANSYLKE
C. acetobutylicum CA_c0957      YGCINMNKNELGVAQQFFLSSNVLFIKLNHYEDIINTFINLGKITLNLKAYNSSCSYFEQ
                                 . : :...:    *    * .:  :*:: .   :: . ::*:.** :. . : .: * ::

C. difficile RstA               AEEILSENKFEDVNIHKELYSKMAYCYIKLDNPNMSLKYIEKINE-IDNRNDRKEDVDIL
C. sordellii ATCC9714_31891     AEEIIEENKLNDVNIKKDLYSNISFCYTQLNKDDDALEYIEKIKN-LDITINCKEEAGLM
C. perfringens CPE1448          AEKIYINNKLTEDLLLGEIYYLISKTYFFIENIQKAINFAFLAKSEFEKVYNKKAYAKSL
C. acetobutylicum CA_c0957      AERVSRDNDIGNDFLIGEIYYYMSLVYFKMNNSNKTIKYCLMAKEKFAELENKKEYARTL
                                **.:  :*.: :  :  ::*  ::  *  ::: : ::::    :. :    : *  .  :

C. difficile RstA               VLKANNMLEIGKYEESRDYFKKALKILESEDNKTGLANVYLTICDVYRKIGETDRVLEYS
C. sordellii ATCC9714_31891     VSKANKLMSVGKYEEAKQQFKTALDLLEKEDNKTGLANVYIRICEVYESIGESEKSLEYS
C. perfringens CPE1448          ELLAEEHAKVGDLESAIAYSKQSLNVYKELEQLDKISSIEDSLGKLFFEFDNIEESFEHY
C. acetobutylicum CA_c0957      LILSKEYSKSGDVDNAIKYSKKALKAFKENDNMFAVADIENELGKLFYEFENIEESFIHL
                                   :::  . *. :.:     * :*.  :. ::   ::.:    : .:: .: : :. : :

C. difficile RstA               QKVYDIKKNDEDEYMMSGLFKIIEAYIDKEDYDLARKYCK-IALASSIKNKNKFNEYKAL
C. sordellii ATCC9714_31891     KKAYEIKRYDEDECSIDVIIKIIQSYIKLNNFEEARKYSK-TALASAIKNKNKYFEYKAL
C. perfringens CPE1448          NRAIKIREENTDSKLLDTLFNLCENHIKTKNMNKCIEILNEIKNKLNVSDVNRLIEYKIL
C. acetobutylicum CA_c0957      NKAKEIRKKTKGHEFIETLCNICENYIKLKDTENSQKVLNEIKENIDGNNVFDILKYYML
                                ::. .*:.    .   :. : :: : :*. :: : . :  :         .:     :*  *

C. difficile RstA               RFYANMYKNQNEVTLAIEYLIKCIKIVSDLGNNRILANLYIDLGQLYSNISKEKE-LEYY
C. sordellii ATCC9714_31891     KYYSEIYKKEDNNKLAIEYLLKCINIIKELNDKKTLADLYIELGKLYSNISKEKE-LEYY
C. perfringens CPE1448          TFRIEVLQ--NNIKEAENILLDTLEIAKRNDMKKKEADISIRLGKFYLELKRDYEAAKYL
C. acetobutylicum CA_c0957      KYRISILN--RSLKEAENILLRALQYAENMELEKEAAEFSVIIGKYYIDNGDDVEAAKYL
                                 :  .: :    . . * : *:  ::  .     ::  *:: : :*: * :    : *  :*

C. difficile RstA               QKGVFMYKNLEIM---
C. sordellii ATCC9714_31891     QKGVCLYQNLEII---
C. perfringens CPE1448          DKGINNLKNLGVIKS-
C. acetobutylicum CA_c0957      NSGIEKFKKIGVLKDL
                                :.*:   ::: ::
```

B

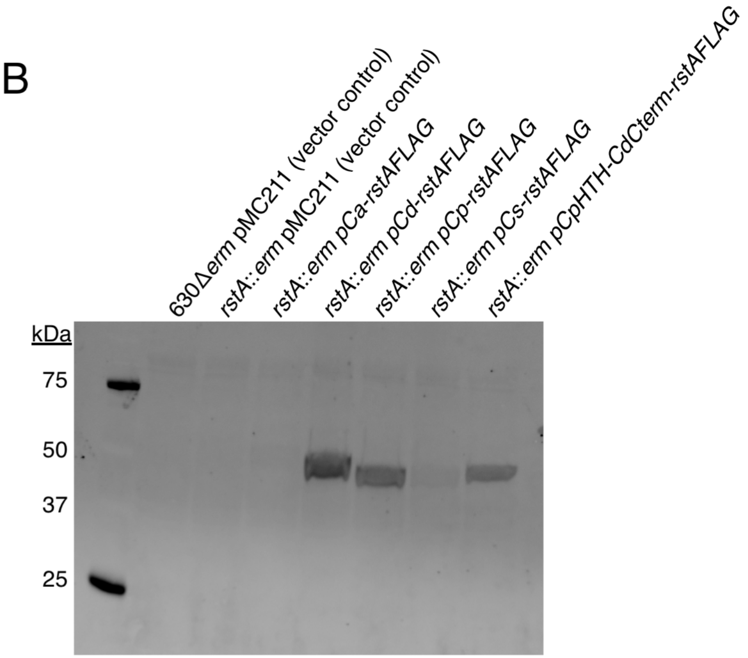

Figure S9. Alignment and stability of RstA orthologs encoded in *C. difficile* 630, *C. sordellii* ATCC 9714, *C. perfringens* S13, *C. acetobutylicum* ATCC 824. (**A**) Multiple sequence alignment performed by EMBL-EMI Clustal Omega tool. The regions denoting the DNA-binding domain (from M1 to Y51), which were used in the RstA *C. perfringens* DNA-binding domain-*C. difficile* C-terminal domains hybrid construct, and the residues deleted in conserved helix-turn-helix motif for the *rstA*ΔHTH-3XFLAG construct are marked. The key is as follows: (*) identical; (:) strongly conserved; and (.) weakly conserved residues. The colors group the amino acid residues based on their physiochemical properties: (red) small, hydrophobic residues; (blue) acidic residues; (magenta) basic residues; and (green) hydroxyl-sulfhydryl- or amine-containing residue. (**B**) Western blot analysis using FLAG M2 antibody to detect recombinant RstA-3XFLAG proteins (~51 kDa) expressed in 630Δ*erm* pMC211 (MC282; vector control), *rstA*::*erm* pMC211 (MC505; vector control), *rstA*::*erm* pMC828 (MC1323; *Clostridium acetobutylicum rstA* [*Ca_C0957*]*), rstA*::*erm* pMC675 (MC1004; *Clostridium difficile rstA*), *rstA*::*erm* pMC829 (MC1324; *Clostridium perfringens rstA [CPE1448*]), *rstA*::*erm* pMC830 (MC1325; *Clostridium sordelii rstA* [*ATCC9714_3891*]) and *rstA*::*erm* pMC798 (MC1257; pP*cprA-rstACpHTHCdCterminal*-3XFLAG) grown in TY medium supplemented with 2 μg/ml thiamphenicol and 1 μg/ml nisin at H_{24}.

Sievers F, Wilm A, Dineen D, Gibson TJ, Karplus K, Li W, Lopez R, McWilliam H, Remmert M, Soding J, Thompson JD, Higgins DG. 2011. Fast, scalable generation of high-quality protein multiple sequence alignments using Clustal Omega. *Mol Syst Biol* 7:539.
