# Supplementary material for: RstA Is a Major Regulator of Clostridioides difficile Toxin Production and Motility
Source: mBio. 2019 Mar 12;10(2):e01991-18. doi: 10.1128/mBio.01991-18 (PMC6414698; doi:10.1128/mBio.01991-18)
Supplement: FIG S10 [file mBio.01991-18-sf010.pdf]

Figure S10.

Promoter	Sequence	Element
P*rstA*	-------TAAAATAAATATAAGTTATAATTATATTA------	-10
P*tcdA*	----ACATTAAAAAAATATAAGATATGTTTACAAATT-----	-35
P*tcdB*	AATTTTTATATTTTATATAGAACAAAGTTTACATATT-----	-35
P*tcdR*(σ^A)	--TGATTTAATTCCAATGTTGTCAAAATTTTCAAATAAATCA	-35
P*tcdR*(σ^D)	-----------ATTAATGTATTCATAATGCATATTTTCAT--	
P*flgB*	--ATATTTAAAAAATTTGTTATTTAT----------------	-35

Figure S10. Alignment of inverted repeats within the promoters of direct RstA targets. The inverted repeats identified by EMBOSS Palindrome Finder (Rice, *et al*. 2000) within each promoter that RstA directly binds are shown. The conserved -10 or -35 elements within each promoter are underlined. The predicted -35 element for PtcdR(σ^D) begins immediately following the sequence shown here. The two nucleotides that are important for RstA binding to P*rstA* DNA are marked in red.

Rice P, Longden I, Bleasby A. 2000. EMBOSS: the European molecular biology open software suite. *Trends Genet* 16: 276–277.
